# Risk of stress/depression and functional impairment in Denmark immediately following a COVID-19 shutdown

**DOI:** 10.1186/s12889-021-11020-3

**Published:** 2021-05-26

**Authors:** Lars H. Andersen, Peter Fallesen, Tim A. Bruckner

**Affiliations:** 1 ROCKWOOL Foundation, Ny Kongensgade 6, 1472 Copenhagen C, Denmark; 2 Swedish Institute for Social Research, Stockholm University, 106 91 Stockholm, Sweden; 3 Program in Public Health and the Center for Population, Inequality and Policy, University of California, Irvine, CA 92697-3957 USA

## Abstract

**Background:**

Existing estimates of the impact of the COVID-19 burden on mental wellbeing come from countries with high mortality rates. This study therefore aimed to investigate the impact of the first COVID-19 lockdown (March–April 2020) on risk for stress/depression and functional impairment in a representative sample of adult individuals in Denmark, which had lower infection rates, and whether the impact of lockdown was heterogeneous across living situation.

**Methods:**

Using a representative, randomly drawn sample from the complete Danish adult population interviewed in March 2 to April 13, 2020 (*n* = 2836) and again in July 2020 (*n* = 1526, 54% retention rate), we study how the imposed lockdown announced March 11 following the onset of the first Danish wave of COVID-19 infections affected mental wellbeing. We use the World Health Organization Five Well-being Index (WHO-5) and the Work and Social Adjustment Scale (WSAS) to capture risk for stress/depression (WHO-5 < 50) and functional impairment (WSAS > 10). Using covariate adjusted ordinary least squares linear probability models and exploiting variation in the timing of responses occurring just before and just after the introduction of lockdown, we compare respondents before lockdown to respondents that answered during lockdown, as well as to answers in re-interviews in July.

**Results:**

In our fully controlled models, we find reduced depressive symptoms among adults immediately after the shutdown, concentrated in adults with children living at home (−.089, *p* < .01 (from pre lockdown baseline .273)). Measures of functional impairment also declined immediately after the March shutdown among adults with children living at home (−.066, *p* < .05 (from pre lockdown baseline .150)). Impairment intensified for the entire sample between March and July (+.199, *p* < .001 (from pre lockdown baseline .248)), but depressive symptoms remained at lower rate in July (−.033, *p* < .05 (from pre lockdown baseline .332).

**Conclusions:**

Findings in Denmark indicate that living with children at home may have, in the short term, buffered the potential mental health sequelae of the COVID-19 shutdown.

**Supplementary Information:**

The online version contains supplementary material available at 10.1186/s12889-021-11020-3.

## Introduction

As of mid-October 2020, more than 90 countries across the world had imposed some form of lockdown in the wake of the COVID-19 pandemic [1]. Lockdowns range in scope and duration, but all imply a degree of social isolation as well as disruption from routine social, educational, and/or work activity. Previous research which predates COVID-19 indicates that, following social isolation and disruptions from work routines, mental wellbeing may decline [2–6], and whether people live together with others or not can be an important stratifying factor [7]. In addition, a review of mental health following more extreme measures of quarantine finds long-term psychological sequelae [8], and studies have found increased levels of depression, stress, and anxiety among several groups, such as singles, females, and people who lost their jobs because of lockdowns [9–11] . Taken together, this literature has raised the concern of a “second pandemic” of morbidity due to mental health problems following COVID-19 [12], and calls have been made for prioritizing high-quality research on the mental health effects of the pandemic [13].

Recent research examines mental wellbeing following COVID-19 and the associated lockdowns. Four population-representative studies—in the UK, the US, Austria, and France— appear in the literature together with a study across eight countries using respondents driven sampling. All studies report worse mental health in Spring 2020 relative to previous years [6, 12, 14–17]. Ettman and colleagues, for instance, find a much greater prevalence of depressive symptoms among adults in the US in April 2020 relative to 2017/2018, just as Sønderskov and colleagues do for Denmark in early April 2020 relative to 2016.

**Fig. 1:**
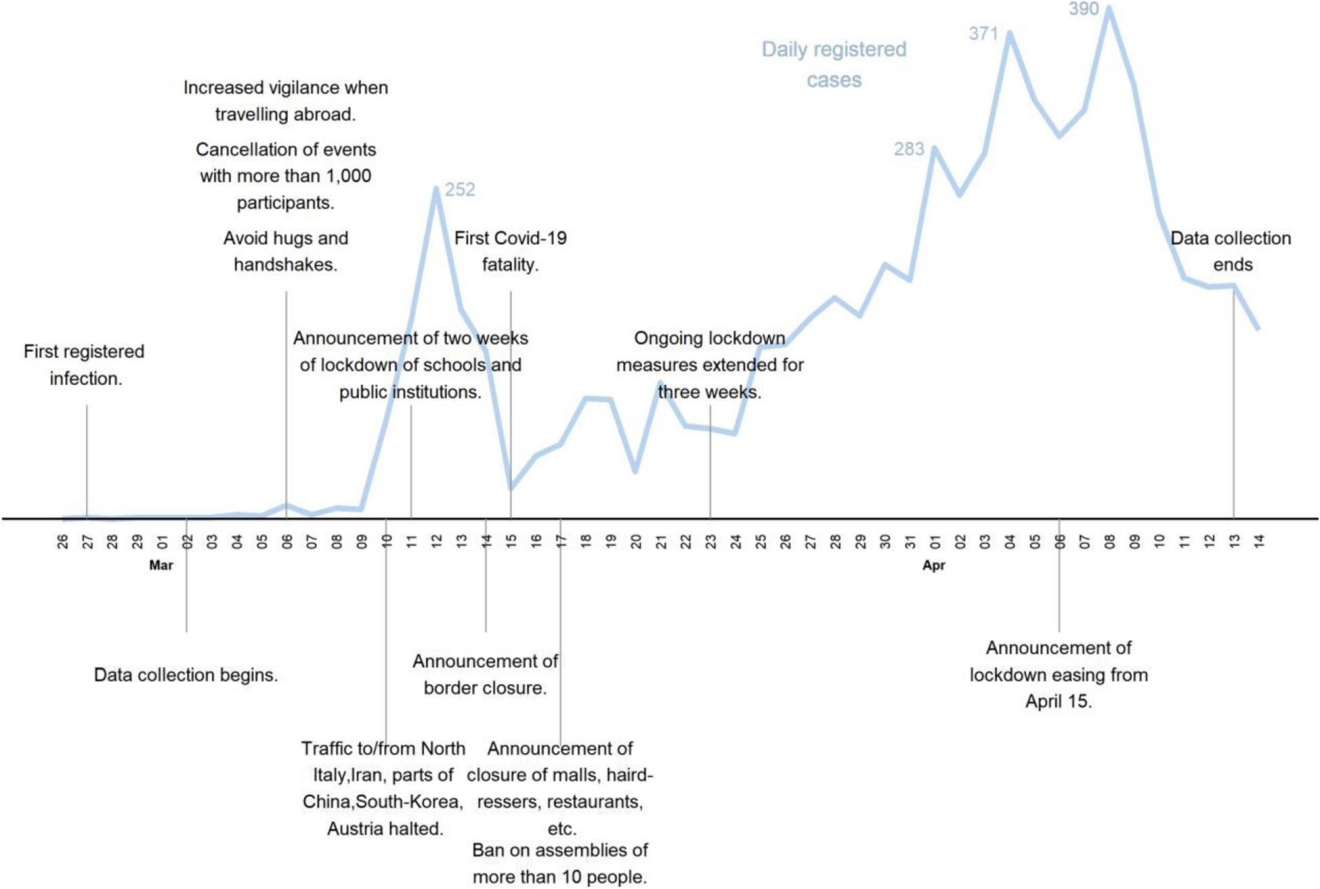
COVID-19 and lockdown development during first wave of data collection, February–April 2020

This work, while important, has two key limitations. First, among the national studies, the UK, the US, and France all rank in the top 15 worldwide in COVID-19 deaths per population as of November 30, 2020 [18]. This circumstance leaves open the question of whether experienced national severity of the pandemic (through media reports [12] or through direct experiences), or the social and work restrictions imposed by a lockdown per se, drive results. Second, none of these studies includes measures of mental health and/or wellbeing immediately *before* the lockdown. The absence of “baseline” mental health information in weeks before the lockdown raises the concern of confounding by trends over time in mental health that coincide with, but are not caused by, the COVID-19 lockdown. Third, unlike for previously studied countries, the Danish lockdown was imposed uniformly and rapidly following the first infections and came into effect before the first Danish registered COVID-19 fatality (see Fig. 1).

We address these limitations and extend prior work by examining mental wellbeing in Denmark, a country that imposed a lockdown in March 2020 but reports a substantially lower COVID-19 burden as of November 30, 2020 (i.e., 14.3 deaths per 100,000 population) than does France, the US, or the UK (i.e., 78 to 87 deaths per 100,000 population) [18]. We also exploit variation in the timing of responses to a nationally representative survey collected in March 2020. On March 11, 2020 – the day the World Health Organization declared the COVID-19 outbreak a global pandemic – Denmark imposed nationwide school closures and the closing of public institutions. Survey responses occurred immediately before and after the date when the first COVID-19 lockdown was ordered and imposed, in a setup not dissimilar to the one applied by Ahrens and colleagues in Germany [19].

We measured mental wellbeing among the adult Danish population through the World Health Organization Five Well-being Index (WHO-5) and the Work and Social Adjustment Scale (WSAS). These scales capture both pre-clinical measures of mental disorder as well as impairment. Further, we re-interviewed the sample in July 2020 when COVID-19 precautions were substantially lessened compared to the lockdown period in March. Given that previous research on some subgroups finds *improved* wellbeing following COVID-19 [19, 20], we specified all tests as two-tailed. We, moreover, explored the relation between the lockdown and mental wellbeing by family structure, given that state-imposed limitations on social activity may affect persons living alone differently than for persons living with family members as suggested by prior research.

## Background

### Lockdown timeline for Denmark

Figure 1 provides a timeline of the Danish COVID-19-restrictions, the number of confirmed cases for March and start of April 2020, and the data collection window for the first wave of the survey [1, 21]. Denmark reported its first confirmed SARS-CoV-2 case on February 27, 2020 [1]. On March 11, Denmark initiated nationwide school closures and the closing of public institutions, as the cumulative number of confirmed infections had increased to 264. Lockdown measures were further strengthened over the following 6 days to include border closures, the closure of restaurants, malls and hairdressers, and general encouragements to work from home. Financial aid packages to businesses and furloughed employees were launched in the same six-day period. Unlike other European countries, Denmark did not introduce curfews, stay-home orders, or mandatory use of masks during the Spring lockdown. The Danish government began easing lockdown measures from April 15, 2020. Lockdown measures were continuously eased over the summer, and not re-introduced until September 2020. Based on this timeline, we defined the beginning of the lockdown measures as March 11, 2020.

We address the mentioned limitations and extend prior work by examining mental wellbeing in this low-COVID-19-burden country. There are three reasons why we still expect to observe effects of lockdown on mental wellbeing in this context. First, witnessing critical events, such as the growth of a pandemic, could impair wellbeing even when the event is not local, as when the 9/11 terrorist attack in the US caused an immediate 16% increase in the incidence rate of trauma- and stressor-related disorders in Denmark [22]. Relatedly, Ahrens and colleagues argue that physical distancing and lockdowns can be seen as “global macro-stressors” [19]. Second, a recent multi-country study found that people experiencing physical symptoms akin to those symptomatic of COVID-19 suffer worse mental health, implying that perceived impact of the pandemic could also be important [23]. And third, experiencing even a minor lockdown order could still affect people’s mental wellbeing, as lockdown for many implies social isolation and little to no (physical) contact with others, and as people had to adjust to new daily schedules including homeschooling and working from home.

## Methods

### Variables and data

To consider how measures of wellbeing and impairment changed following the imposed lockdown, we used a representative survey carried out by Statistics Denmark on behalf of the Capital Region of Denmark’s Mental Health Services during March–April 2020 (see Additional file 1 for a flow chart of the data collection). Using a random draw from the present population database of all Danish residents aged 18 and above, Statistics Denmark initially contacted 8300 people through personal digital postboxes that are linked directly to people’s unique social security numbers and used for communications between Danish residents and governmental institutions. All people were contacted at the same time in early March and could respond to the survey when convenient within the survey period (through to mid-April). Respondents answered through computer assisted web interviews (CAWI), with those initially failing to respond receiving prompts by message to their digital postbox. The response rate to the first wave of the survey was 34% (*N* = 2836); 1127 respondents completed the survey prior to the lockdown announcement and initiation on March 11, and 1709 respondents completed it after March 11. These numbers reflect respondents who provided valid responses to all items of our dependent variables. Respondents who completed the survey before and after March 11 were generally alike across the background characteristics, although the proportion of respondents age 60+ decreased and the proportion of respondents with children living at home increased slightly (see Additional file 1).

With permission from the Capital Region of Denmark’s Mental Health Services, we then carried out a follow up survey in July 2020, where the same respondents were re-interviewed. Again, invitations were sent out to everyone at the same time, early July, and respondents could respond to the survey when convenient throughout the month. Of respondents participating in the first wave, 1526 (54%) also participated in the second wave collected in July 2020, for whom we thus have repeated observations. Younger respondents and respondents with children living at home were less likely to participate in the second wave, as were respondents who experienced significant functional impairment in early March (which will likely cause us to underestimate a potential increase in functional impairment from March to July), but respondents in the second wave generally resemble respondents in early March (see Additional file 1). Answers to the survey can be linked at the individual level with administrative data from Statistics Denmark. In addition to survey data on mental wellbeing and functioning, our data therefore contain information on age, gender, living arrangement (single/in a relationship), whether respondents were living with any children in the home, region of residence (Nomenclature of Territorial Units for Statistics, level 2 ([NUTS-2]), and employment status (employed, unemployed, outside the labor force).

To capture wellbeing and experienced functional impairment, the survey included two validated measures—the WHO-5 and the WSAS—that both have distinct clinically relevant threshold values. A review finds that the WHO-5 remains among the most widely used questionnaires assessing subjective psychological well-being [24]. Since its first publication in 1998, the WHO-5 has been translated into more than 30 languages and has been used in research studies all over the world. The WHO-5 is a sensitive and specific clinical screening tool for risk of stress and depression that uses five items to capture risk of depression measured between 0 and 100, with each scale contributing 0–20 points. For this measure, 0 indicates the most severe depressive symptoms and 100 indicates no symptoms. The WHO-5 has strong construct validity as a unidimensional scale [24]. For Denmark, the population norm is established as 70 for adults [25]. For the WHO-5 index, we (consistent with work in clinical settings) used the established cutoff point at 50 to create a binary category for whether a respondent is at risk for depression and stress (i.e. WHO-5 < 50, [24]).

The WSAS is a functional impairment measure designed to measure a patient’s perceived functional impairment following health problems across five items [26]. Mundt and colleagues’ paper (*British Journal of Psychiatry* 2002) of the WSAS finds that this instrument is a reliable and valid measure of impaired functioning. Although not originally intended for non-clinical populations, the WSAS displays valid psychometric properties across different patient populations that cover both mild and more severe (psycho-)somatic [27–29] and mental health [26, 30–32] conditions. Furthermore, the WSAS captures a dimension of impairment distinct from depression [32]. Although not validated in a Danish version, it has previously been used both in Danish clinical and research settings [30]. WSAS measures impairment on a scale from 0 (no impairment) to 40 (most severe impairment). Given that we study a non-clinical sample, we use the cutoff point at a WSAS score of 10, with any score above 10 indicating at least significant functional impairment with or without additional psychopathologies (WSAS > 10 [26]).

### Analytical strategy

Our analytical strategy exploits the fact that data collection took place across the announcement and initiation of lockdown in Denmark in March 2020. First, we use ordinary least squares linear probability models to compare the outcomes between respondents who answered the survey before and after lockdown began. We adjust all models for gender (indicator variable for female, male as reference category) and age (indicator variables for 10-year age groups with Ages 40–49 as reference category) and use t tests to evaluate differences between respondents who answered the survey before and after lockdown began. Next, in fully adjusted regression models, we control for NUTS2-region of residence (Capitol Region of Copenhagen as reference category), labor market status (employed, unemployed, outside the labor force (reference)), relationship status (single, married/cohabiting (reference)), and whether respondents had children at home (no children at home as reference category), and again use t tests to evaluate the statistical significance of differences between respondents who answered the survey before and after lockdown began.

The impact of lockdown could differ according to the home environment. Persons living with family, for instance, may experience relative more social interaction than would persons not living with family during the imposed lockdown. To explore this possibility, we performed sub-sample analyses that compare single individuals to individuals who are living with a partner, as well as sub-sample analyses that compare people living without children in the home to people living with children in the home, and test for differences between subgroups using Chow-tests (which test whether regression coefficients are different for split data sets; in our case, we split the data by family type).

In addition, to fully leverage our data structure with re-interviews in July 2020, we then compared the reported levels of risk of depression and stress and significant functional impairment measured prior to lockdown in March (we thus drop respondents who responded during lockdown) to the levels experienced by the same persons in July accounting for repeated measures of the same individuals with clustered standard errors. The latter exercise captures the development in the outcomes across the first wave of COVID-19 in Denmark. Here, we again adjust for control variables in the regression but because we use within-individual variation across survey waves this inclusion is not essential (e.g., the age of the individual does not change substantially from March to July). All calculations were carried out using Stata 15/MP.

We then performed several robustness checks. First, we evaluated whether our choice of thresholds in the outcome variables (WHO-5 < 50 and WSAS > 10) affect inference. Second, because our sample is not fully identical to the Danish population on characteristics such as gender and age, we replicate main results using population weights provided by Statistics Denmark instead of adjusting for variables. Third, selective survey participation across the lockdown in March and across the two survey waves (if, for example, people who experience increased mental distress due to the lockdown are less likely to participate than before the lockdown or they are more likely to not respond to survey wave 2) could invalidate our results (Additional file 1 showed some sign of such selection from wave 1 to 2, although not to any discernable degree from early to late March, on observed characteristics). To address this, we use the within person changes in response between March and July. As there was very little change in lockdown measures in July, all returning respondents recompleted the survey under identical lockdown circumstances. If our main pattern of results between respondents who answered prior to and during lockdown in March persist once we take into account individual change up to the post-lockdown July wave, it would indicate results are robust to differential selection in the first wave of response across the lockdown period, and results would thus at least be internally valid. Fourth, the items of the WHO-5 ask respondents to consider their experiences during the preceding 2 weeks. To account for the possibility that respondents answering within 2 weeks after lockdown have to consider both time before and after lockdown, as a robustness check we weighted answers given in the 2 weeks after lockdown with the amount of time since the lockdown announcement. If people considered a full two-week horizon it would mean that our main estimates of the impact of the lockdown order in March will be biased toward zero—that is, our main estimates would be conservative. Last, because our main results focus on respondents with children living at home, and because there may be different requirements and worries associated with having children at different ages at home, we checked whether results differ by age of the children (which we obtained from the general registers).

## Results

Table 1 describes the sociodemographic characteristics of the survey participants and the population. Respondents (*n* = 2836) are similar to the broader Danish adult population in terms of geographical region and socioeconomic status. We observe some dissimilarities for gender and age, which we therefore control for in all reported results. Over half (54%) of participants in the first survey wave in March 2020 completed the survey in July 2020 (Additional file 1). In addition, during the first survey wave, 40% completed the survey before the lockdown (March 11th), and 60% completed the survey after the lockdown, which permits adequate sample size to estimate mean levels of depressive symptoms and functional impairment during these two distinct periods in March.

**Table 1:** Descriptive statistics of sociodemographic characteristics of survey participants and all the 18–79-year-old people in Denmark

Variable	Survey		Population		T-test	*p*-value
	M	SD	M	SD		
Male	0.449	0.497	0.500	0.500	−5.399	< 0.001
Female	0.551	0.497	0.500	0.500	5.399	< 0.001
Ages 18–29	0.138	0.345	0.205	0.404	−8.839	< 0.001
Ages 30–39	0.109	0.312	0.155	0.362	−6.835	< 0.001
Ages 40–49	0.157	0.364	0.172	0.378	−2.112	0.035
Ages 50–59	0.226	0.418	0.183	0.387	5.844	< 0.001
Ages 60–69	0.205	0.404	0.153	0.360	7.715	< 0.001
Ages 70–79	0.165	0.371	0.131	0.337	5.356	< 0.001
Singles	0.301	0.459	0.353	0.478	−5.714	< 0.001
Couples	0.699	0.459	0.647	0.478	5.714	< 0.001
No children at home	0.675	0.469	0.632	0.482	4.727	< 0.001
Children at home	0.325	0.469	0.368	0.482	−4.727	< 0.001
No. children age 0–2	0.042	0.253	0.054	0.234	−2.667	0.008
No. children age 3–5	0.070	0.311	0.078	0.291	−1.351	0.177
No. children age 6+	0.397	0.780	0.439	0.807	−2.749	0.006
Northern Jutland	0.104	0.305	0.102	0.303	0.311	0.756
Central Jutland	0.245	0.430	0.227	0.419	2.278	0.023
Southern Denmark	0.208	0.406	0.209	0.407	−0.096	0.924
Capitol	0.295	0.456	0.318	0.466	−2.642	0.008
Zealand	0.148	0.356	0.144	0.351	0.629	0.529
In Job	0.652	0.476	0.643	0.479	1.069	0.285
Unemployed	0.019	0.138	0.021	0.144	−0.702	0.483
Outside labor force	0.324	0.468	0.335	0.472	−1.251	0.211
N	2836		4,359,539			

No. children (top coded at three children) and labor market status (In Job, Unemployment, and Outside labor force) are measured from the general registers during the latest available data year (2019)

Bivariate comparison of our outcomes across the lockdown do not reach conventional levels of statistical detection. Still, adults interviewed after the lockdown in March have a slightly lower prevalence of depression and stress when compared to those interviewed before the lockdown (i.e., 20% vs. 22%, see Additional file 1), and this finding is statistically detectable when controlling for age and gender (Additional file 1) yet does not reject the null in the fully adjusted model that controls for additional individual covariates, such as household structure (to which we return; see Additional file 1). If we expand the data to include respondents with valid responses to the WHO-5 items but who had not responded to the WSAS, the decline becomes statistically detectable (i.e., 20% after the lockdown and 23% before, *p* < .05, *N* = 3110). Functional impairment scores from the WSAS also show a slight decline in late March relative to pre-lockdown (i.e., 17% vs. 16%, see Additional file 1), but this difference does not reach conventional levels of statistical detection when we control for age and gender (Additional file 1).

**Fig. 2:**
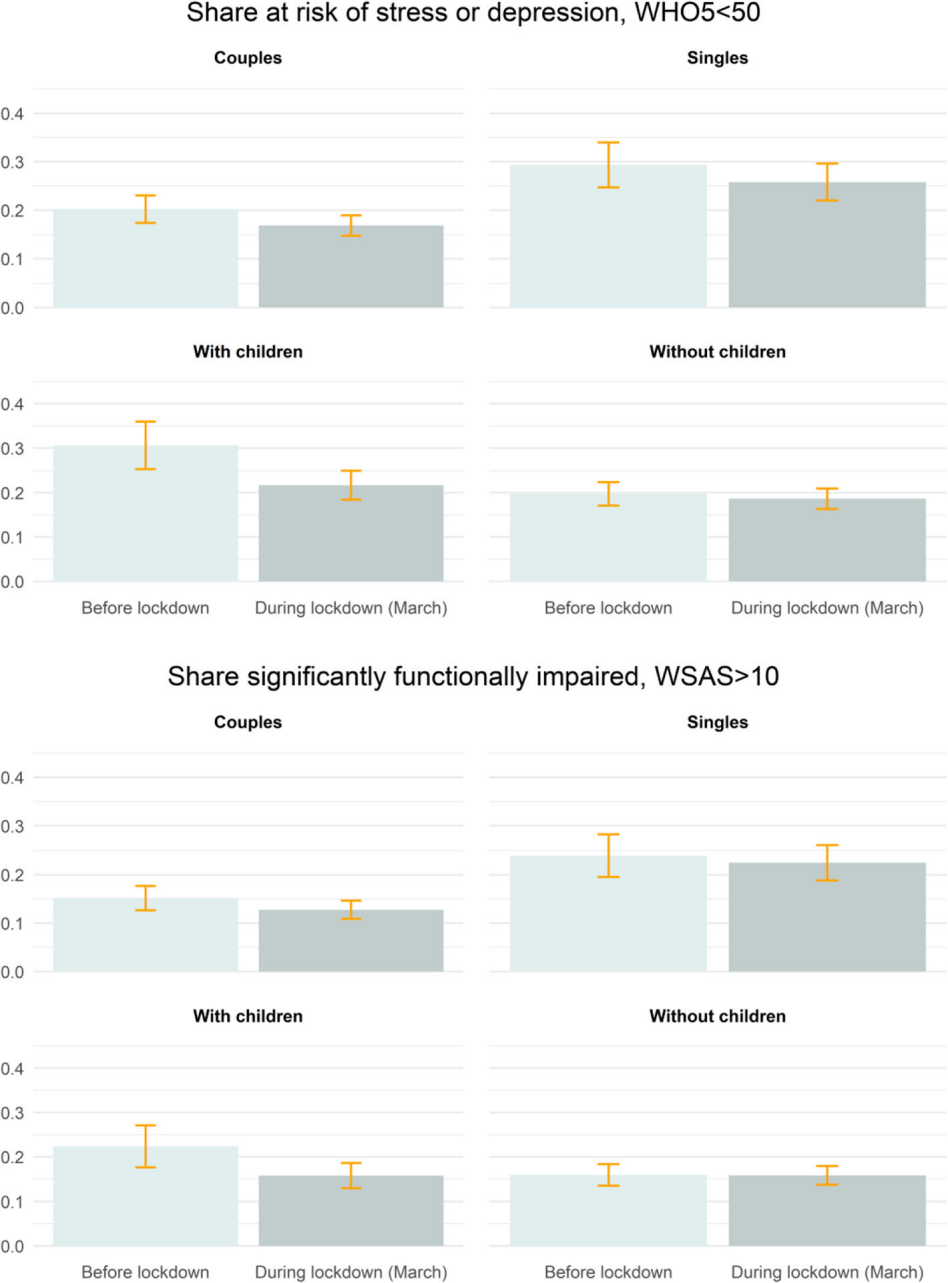
Share with outcomes above clinical threshold of WHO-5 and WSAS by data and household structure

Following the state-imposed limitations on social activity, adults may have relied more on family members for social interaction than they did before the lockdown. Persons living alone, however, may have experienced fewer interactions during the lockdown, which implies the possibility of heterogeneous impact of the lockdown orders. We therefore classified the sample by cohabitation status, and then by whether the adult respondent reported children living at home. Of these subgroups, only adults with children living at home show a lower prevalence of depressive symptoms (upper panel of Fig. 2) in late March (i.e., 17% vs. 32% in early March; *p* < .01, see Additional file 1 for adjusted regression results). Using a Chow-test, we found that the decrease for adults with children in the home compared to adults without children in the home is larger to statistically detectable degree (*p* < .05). All other subgroups report no difference in depressive symptoms between early to late March.

The time course of WSAS functional impairment scores largely coheres with that of the subgroup trends for depressive symptoms. Adults with children living at home show a lower prevalence in functional impairment in late, relative to early, March (lower panel of Fig. 2; *p* < .05, see Additional file 1). Again, using a Chow-test, we found that the decrease for adults with children in the home compared to adults without children in the home is larger to statistically detectable degree (*p* < .05). The lower prevalence of functional impairment among adults with children living at home remains relatively constant across WSAS sub-domains of work, social, and home functioning (Additional file 1). We, by contrast, find no difference in functional impairment scores among other subgroups when comparing pre- vs. post-lockdown periods in March (lower panel of Fig. 2 and Additional file 1).

Lockdown restrictions eased on April 15th. We examined whether depressive symptoms and functional impairment differed among respondents several months later—arguably once COVID-related social, economic, and institutional conventions in Denmark stabilized for a while. We restricted the study sample to persons who completed the survey in early March (i.e., pre-lockdown) and again in July 2020 (Additional file 1 show results including late March respondents). In aggregate, depressive symptoms among these adults fell, but functional impairment rose, in July relative to early March (*p* < .05 for both tests—see Additional file 1).

**Fig. 3:**
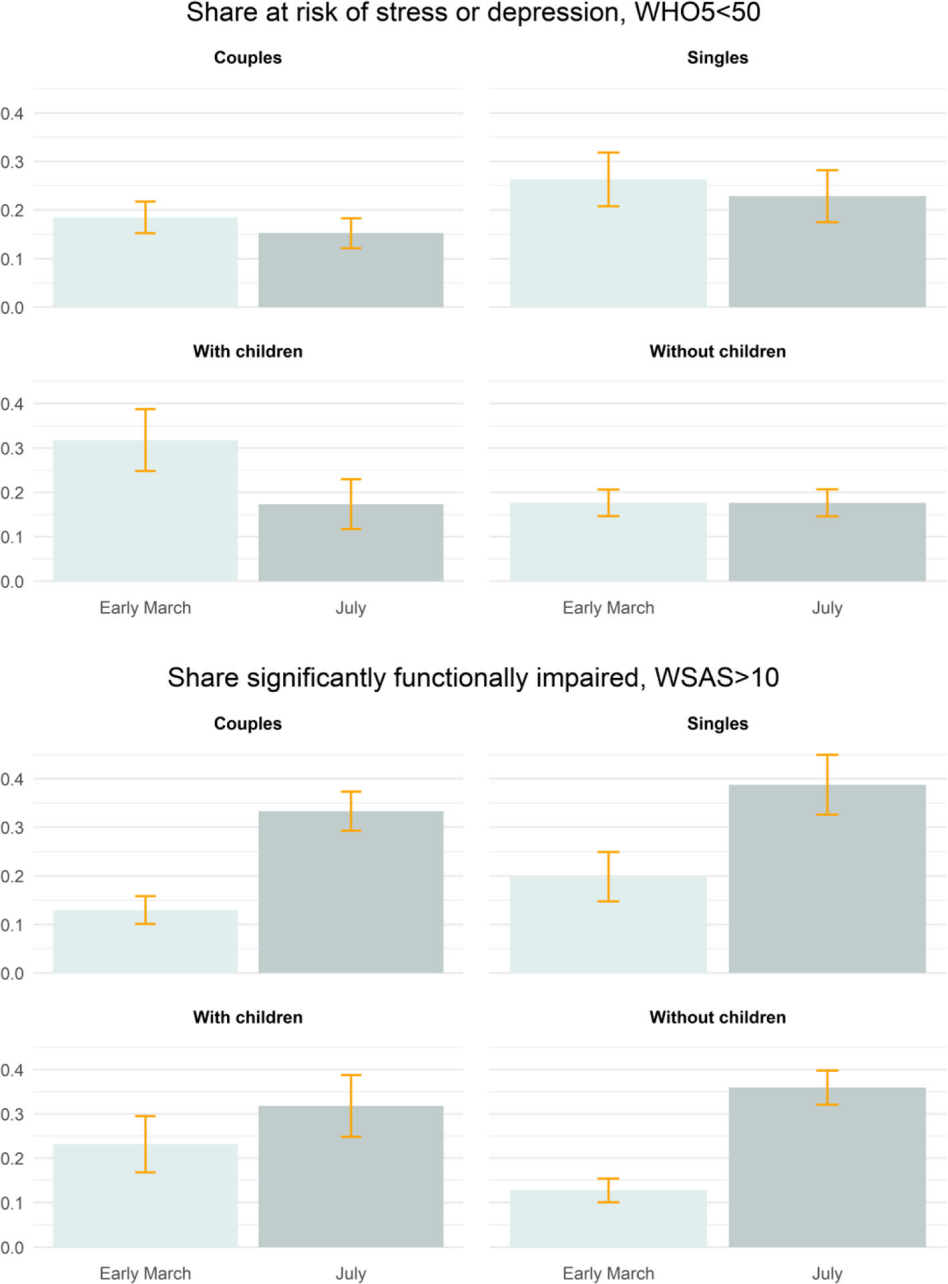
Share with outcomes above clinical threshold of WHO-5 and WSAS among repeat respondents by household structure

When disaggregating the July responses by family structure, only adults with children living at home and adults in couples reported a reduction in depressive symptoms in July relative to early March (upper panel of Fig. 3; *p* < .05, see Additional file 1). By contrast, adults with no children at home as well as singles show no change in depressive symptoms over time (*p* = .713 and *p* = .081, respectively, see Additional file 1). Functional impairment, however, increased in July for all groups, albeit to a much greater extent for adults without children living at home (i.e., 13 to 35% in July; see lower panel of Fig. 3 and Additional file 1). The increase in functional impairment in July among adults with children living at home was much lower (but still statistically significant, *p* < .05).

The results from our robustness checks do not raise concern over the validity of our main results. First, modifying the thresholds used to define depressive symptoms and functional impairment did not substantially change results (see Additional file 1). Second, using statistical weights provided by Statistics Denmark instead of controlling for covariates did not affect inference (see Additional file 1). Third, relying on within-individual differences in the outcomes pre- and post-lockdown in March compared to July answers did not change main results (see Additional file 1). Fourth, down-weighting “exposure” to the lockdown among respondents who participated on March 12th to 25th, as described in the Methods section, did not affect inference (see Additional file 1; we ran the same robustness check for the WSAS and again found results similar to the main Tables (Additional file 1)). Results from our last robustness check (focusing on age of children) shows that our main results for respondents with children living at home are robust across age of the children when focusing on the risk of depression or stress, but that the early to late March decrease in the proportion experiencing significant functional impairment is driven by respondents with children older than 6 years (see Additional file 1).

## Discussion

Existing population-representative estimates of the impact of the COVID-19 burden on mental wellbeing come from countries with high mortality rates. With this study, we contribute to the field by providing estimates from Denmark which had a comparatively low COVID-19 burden but imposed a substantial set of societal restrictions. We exploit the unique timing of a population-based behavioral survey to examine whether a COVID-19 related shutdown preceded an acute change in depressive symptoms and functional impairment. Contrary to reports in most other countries, we find *reduced* depressive symptoms among adults immediately after the shutdown. This result aligns with findings showing a decrease in PTSD symptoms (and no change in stress, anxiety, and depression) during the first 4 weeks from COVID-19 outbreak in China (although the decrease in that study was not statistically nor clinically significant) [33]. And importantly, our results also align with results from Germany, Denmark’s neighboring country, where Ahrens and colleagues document significant improvements to mental wellbeing during lockdown (although they document heterogeneity across groups in the data) [19]. Last, our appear to also be in line with other for Denmark, although mental wellbeing (also measured using the WHO-5) in that study is observed only after the lockdown, from early to late April (i.e., that study has no pre lockdown "baseline" but still documents improved mental wellbeing from early to late April) [17].

The reduction we observe, moreover, concentrates in adults with children living at home. Measures of functional impairment also decline immediately after the March shutdown, but only among adults with children living at home. Again, this finding aligns with results from Germany, where daily hassles decreased during the first 8 weeks after lockdown, likely driven by less commuting and the like [19]. Findings in Denmark indicate that living with children at home may have, in the short term, buffered the potential mental health sequelae of the COVID-19 shutdown. If others replicate our work, strengthening the type of social support that already seems to be present in families may serve as one potential avenue for minimizing the mental health sequelae of extended COVID-19 shutdowns.

Raabe and colleagues’ survey of scientists in three European countries coheres with our findings in that they report improved wellbeing immediately after the COVID-19 lockdown [20], as do results from Ahrens and colleagues for one region in Germany [19]. Similarly, Mari and colleagues find results that mirror ours across residential patterns, although they are limited to studying Italians during lockdown [16]. Also, Tee and colleagues show that some family patterns (i.e., having grown-up children) may reduce the psychological impact of the pandemic in the Philippines [34]. In contrast, a multinational study using data collected late March to early April 2020 generally find that families report the most stress during lockdown [35], but these results may simply reflect differences already existing prior to the pandemic (as our results also suggest). Whereas we hesitate to draw population-based lessons from the select survey of well-educated scientists in Raabe et al.’s study [20], the authors note that strong security of employment may have contributed to their short-term satisfaction with a slower pace and a flexible work-life organization. This financial security may be similar to the situation of most Danish households during the COVID-19 pandemic. Furthermore, social cohesion may increase following adverse events given that shared adversity can connect individuals to a broader goal and purpose than before the event [36, 37]. This social cohesion explanation seems consistent with reports of fewer than expected suicide deaths immediately following the first set of COVID-19 restrictions in Germany and Japan [38, 39]. We note, however, that this explanation is necessarily post hoc and requires further refinement and testing before being considered as anything other than informed speculation. We also point out that the reductions in depressive symptoms among Danes appear confined to adults living with children.

Whereas adults living with children show reduced depressive symptoms in July (relative to pre-shutdown), they are more likely to report significant functional impairment in July. We suspect that, as they habituate to the reality of a prolonged COVID-19 pandemic, the ability to flexibly balance work, family, and social expectations may become strained. Other research also shows that upon returning to workplaces after lockdown, the availability of organizational measures (including significant improvement of workplace hygiene) to tackle COVID-19-related challenges are associated with less severe psychiatric symptoms, which could also explain some of our findings [40]. Interestingly, of any subgroup, adults living with children show the lowest rise in significant functional impairment in July 2020. This result should encourage further investigation, in both Denmark and elsewhere, of elements of family life that may benefit social connectivity and general mental health functioning during COVID-19.

Strengths of our study include the population-based nature of the survey, the use of two different measures of mental wellbeing and functioning, and the fact that survey responses fall immediately before and after the announced lockdown. Limitations involve the fact that the March comparisons of mental wellbeing before and after the lockdown examine serial cross-sections rather than a panel. We, however, controlled for compositional changes of the panel in our analyses. The WHO-5 also asks about 14-day recall of depressive symptoms, which may have biased pre- vs. post March 11 responses towards the null. We, however, controlled for this circumstance using a weighted analysis as a robustness check; findings, moreover, rejected the null, which precludes a type II error. Also, we cannot rule out the possibility of seasonal confounding in that late-March coincided with Spring and better temperature than in early March. This seasonal confounding, however, could only explain the distinct nature of the subgroup responses – in which depressive symptoms and functional impairment fall in late-March only among adults with children but not among adults living alone – if it affects only adults with children and correlates with COVID-19-related societal restrictions (but are not caused by them). Lastly, because we only focused on depressive symptoms and functional impairment, our study cannot address changes in other mental disorders (e.g., suicidal ideation, bipolar disorder, anxiety) following COVID-19-related societal restrictions. These disorders, although strongly correlated with depression, warrant further research and may respond distinctively to COVID-19 related societal restrictions.

Our findings diverge from previous population-based reports in the UK, the US and France. This circumstance could arise for several reasons. First, Denmark underwent a much less severe COVID-19 pandemic in Spring 2020 than did these countries, as measured by overall cases or deaths per population. Danes, therefore, may not have had to contend as heavily with the associated fear and anxiety of COVID-19-related morbidity as did other countries. Second, Denmark’s strong social safety net largely protects adults and families against large financial “shocks” that appear more common in other countries (e.g., the US) when adults lose jobs [41]. Third, the work expectation for adult Danes with children, when the school closures occurred in March, may have been tempered in the short term. As a result, home life with children (at least in the early weeks of the lockdown) may have promoted social interaction and reduced the risk of depression without imposing additional work strain. Future work may want to explicitly consider these important country-level differences when determining what components of the COVID-19 pandemic—the morbidity, the social and educational disruptions, the loss of work—affect changes in mental health and wellbeing. Such work would appear to be critical not only for design of future public health efforts to enhance resilience and recovery, but also for development of theory concerned with collective behavioral responses to adversity.

## Supplementary Information

**Additional file 1.**

**Additional file 2.**

## Data Availability

Data may be obtained from a third party and are not publicly available. The data used in this study have been made available through a trusted third party, Statistics Denmark. Due to privacy concerns, the data cannot be made available outside the hosted research servers at Statistics Denmark. University-based and private Danish scientific organisations can be authorised to work with data within Statistics Denmark. Such organisations can provide access to individual scientists inside and outside of Denmark. Requests for data may be sent to Statistics Denmark: http://www.dst.dk/en/OmDS/organisation/TelefonbogOrg. aspx?kontor = 13&tlfbogsort = sektion or the Danish Data Protection Agency: https:// www.datatilsynet.dk/english/the-danish-data-protection-agency/contact/. The authors document and make available all code needed to reproduce the findings in the study (see Additional file 2).
