# Supplementary material for: Risk of stress/depression and functional impairment in Denmark immediately following a COVID-19 shutdown
Source: BMC Public Health. 2021 May 26;21:984. doi: 10.1186/s12889-021-11020-3 (PMC8149922; doi:10.1186/s12889-021-11020-3)
Supplement: Supplementary file 1 — Additional file 1. [file 12889_2021_11020_MOESM1_ESM.docx]

## Supplementary material to “Risk of Stress/Depression and Functional Impairment in Denmark Immediately Following a COVID-19 Shutdown”

Figure A1. Flow chart of the survey collection.


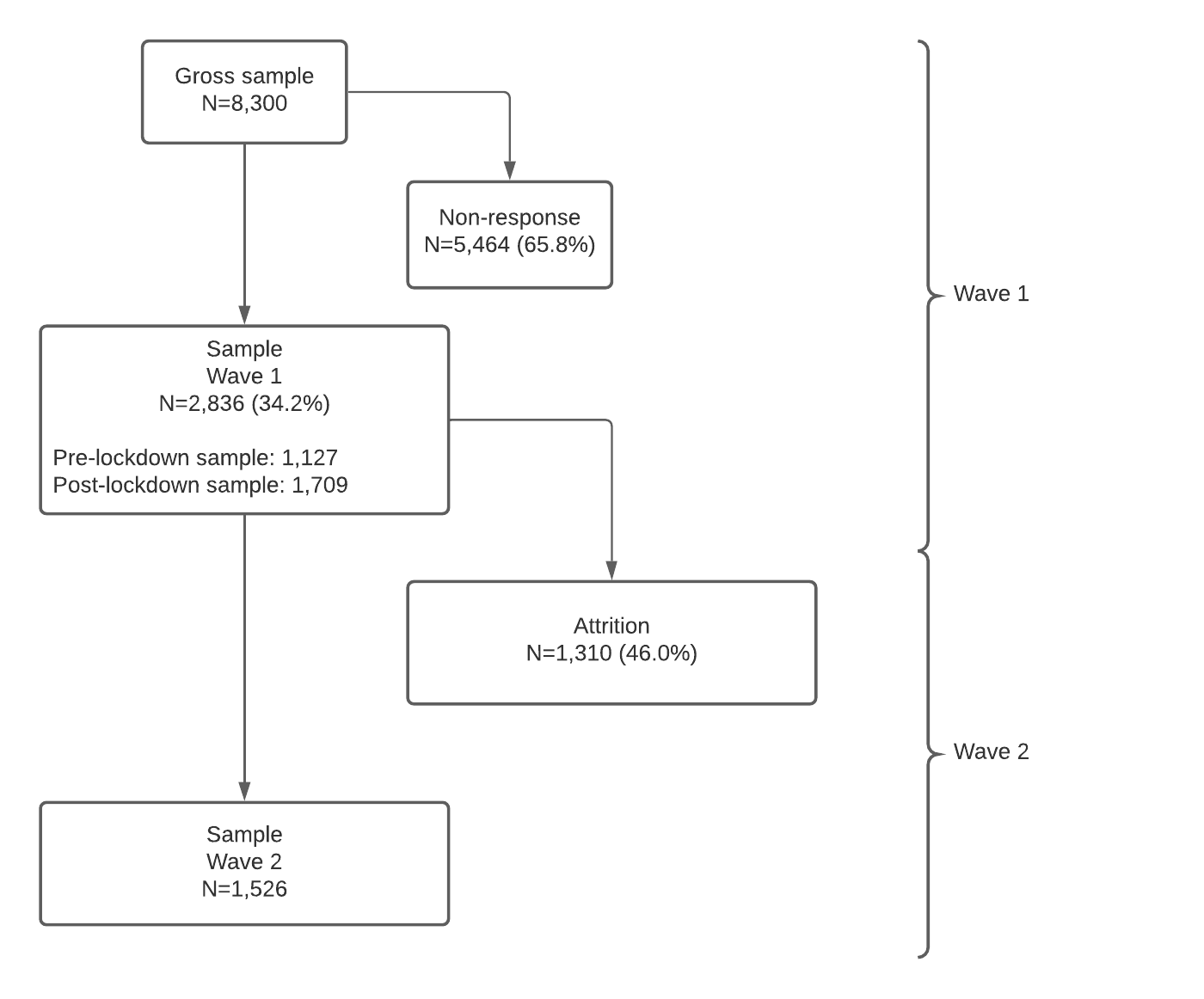


Note: Sampling and interviews performed by Statistics Denmark (DST Survey).

Figure A2. Mean score on WSAS subdomains across time. Only respondents with children living at home.


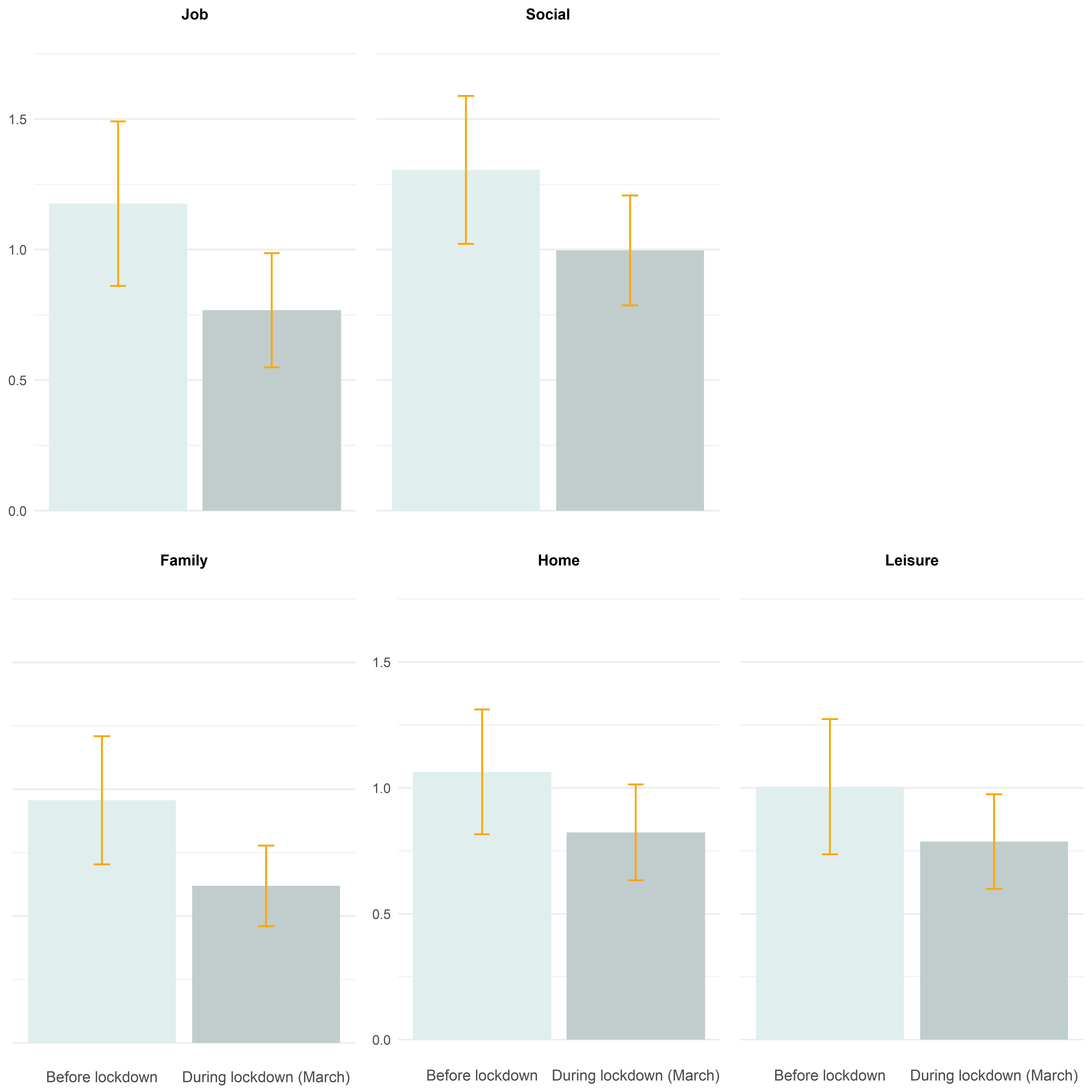


Figure A3. Proportion of respondents above clinical thresholds of WHO5 and WSAS by time
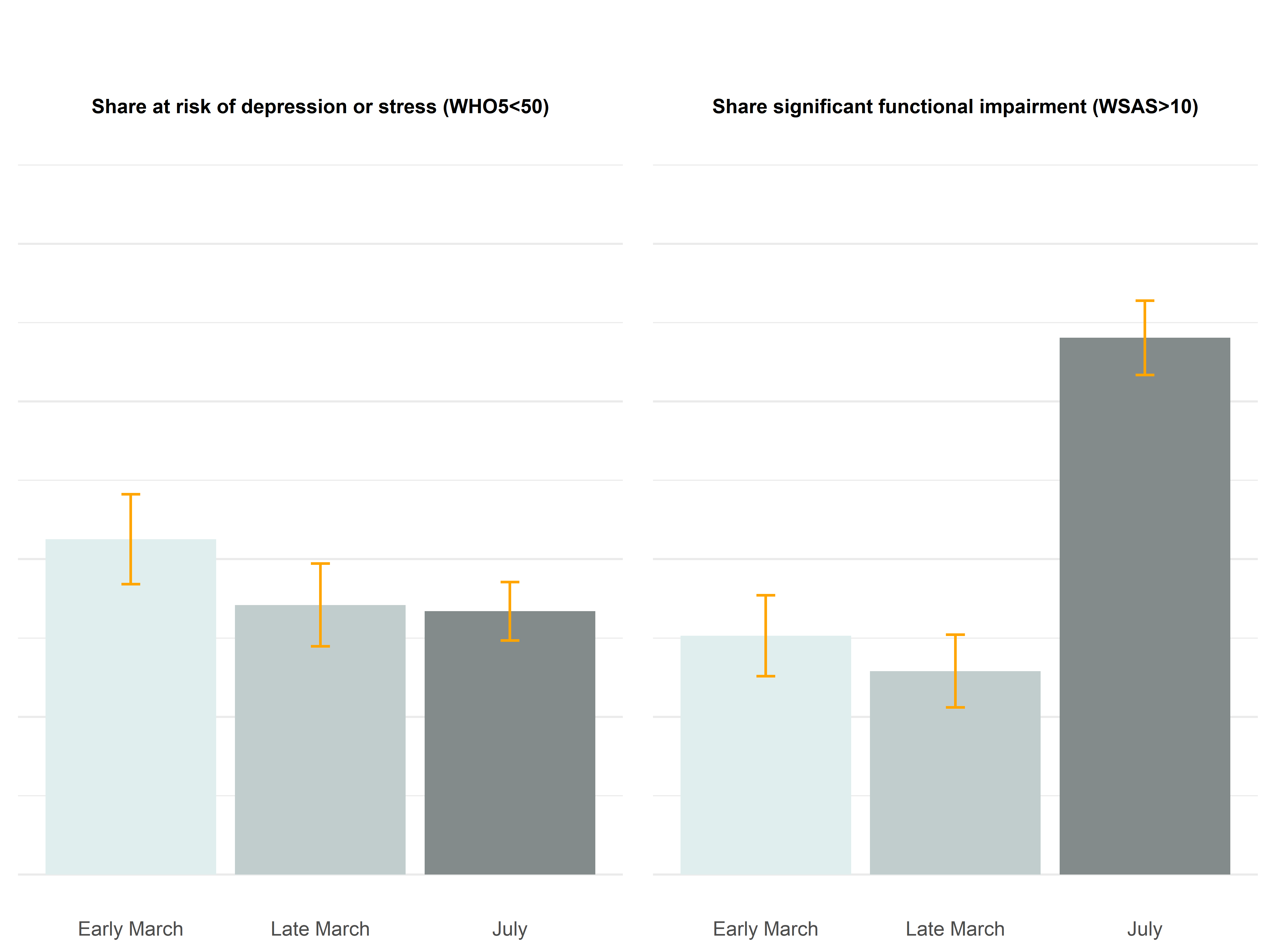


Figure A4. Proportion of respondents above clinical thresholds of WHO5 by time and household structure
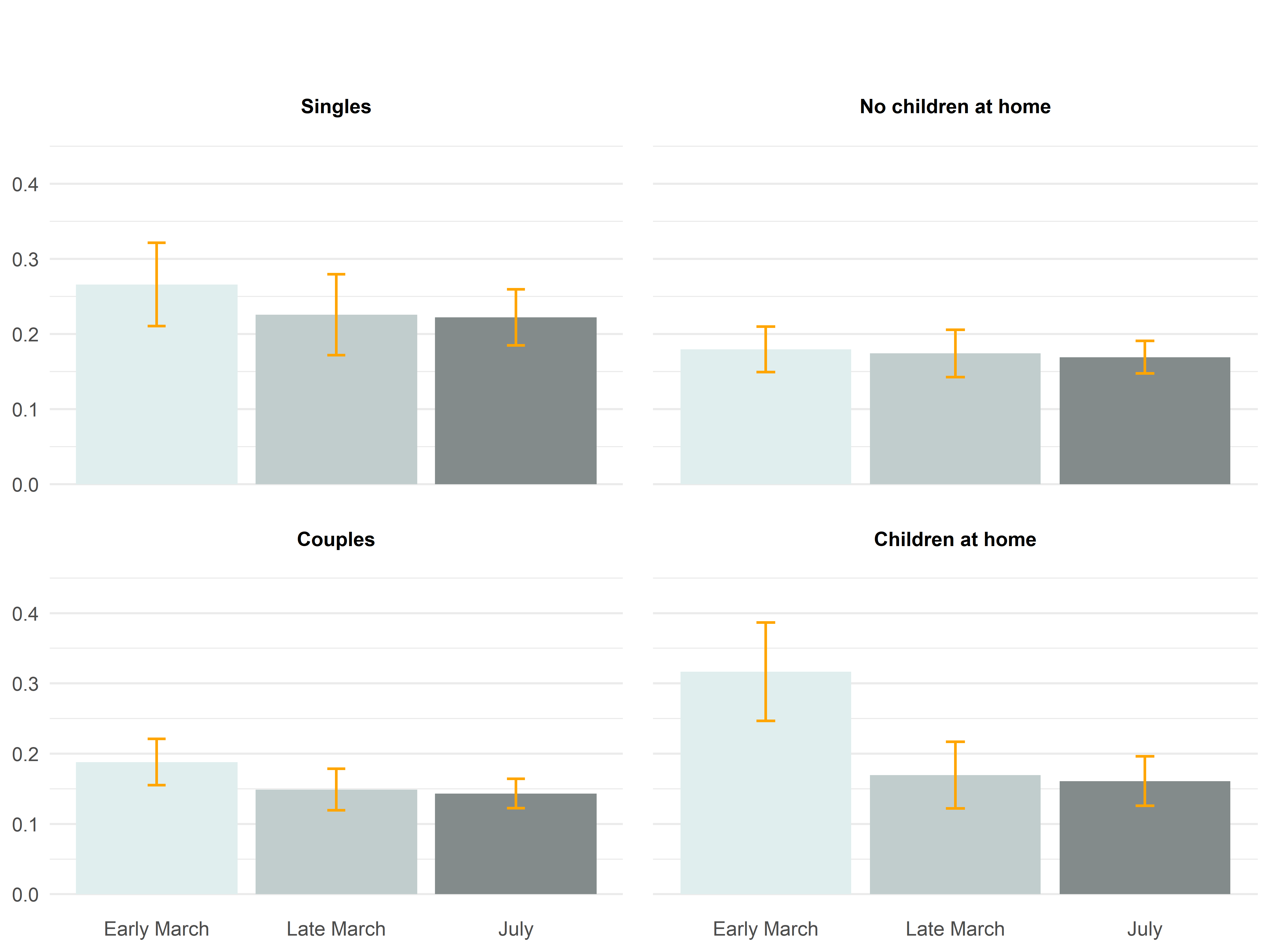


Note: Including respondents in late March.
Figure A5. Proportion of respondents above clinical threshold of WSAS by time and household structure
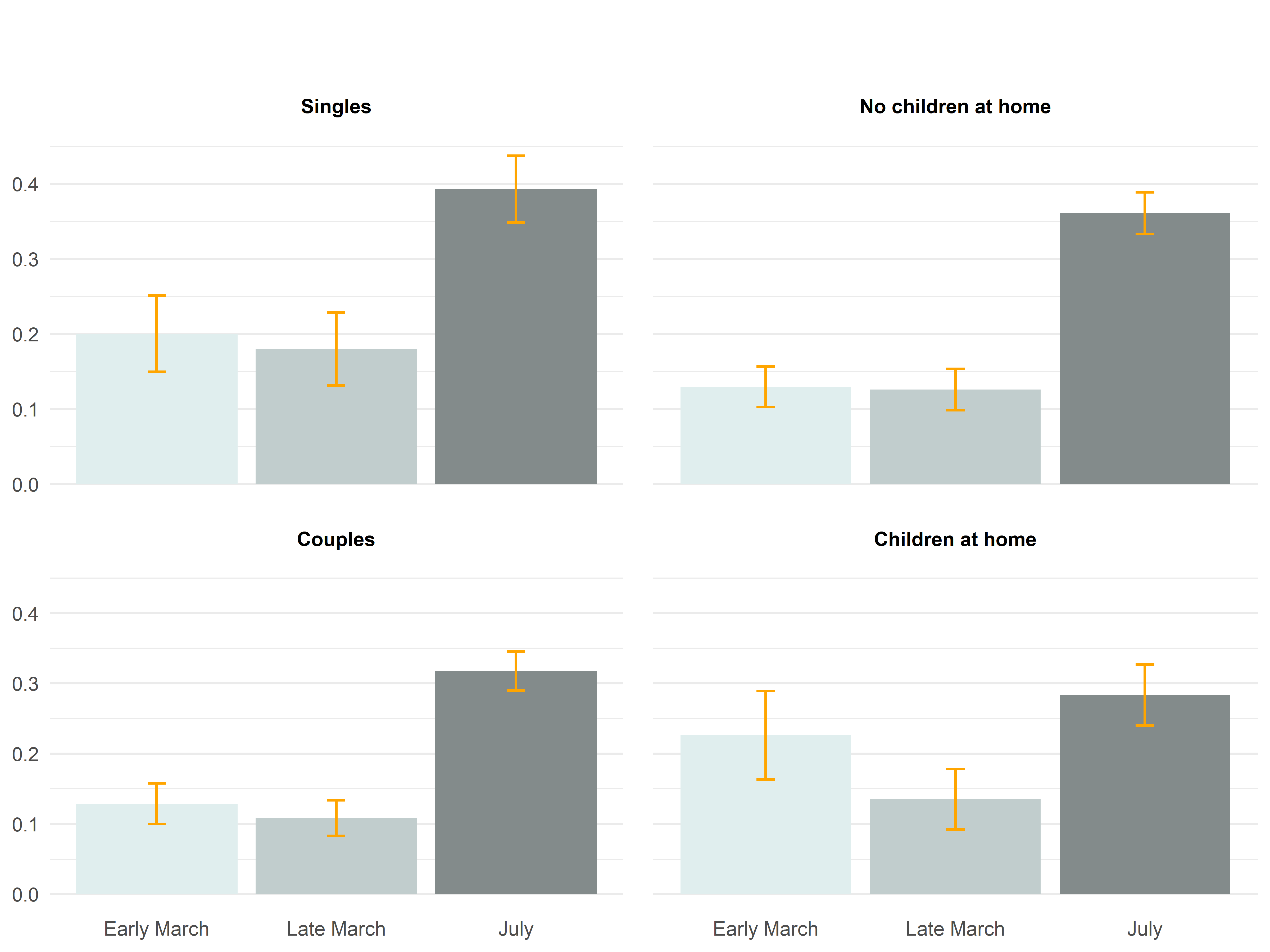


Note: Including respondents in late March.

Figure A6. Main results using population weights from Statistics Denmark instead of statistical controlling for observable characteristics.


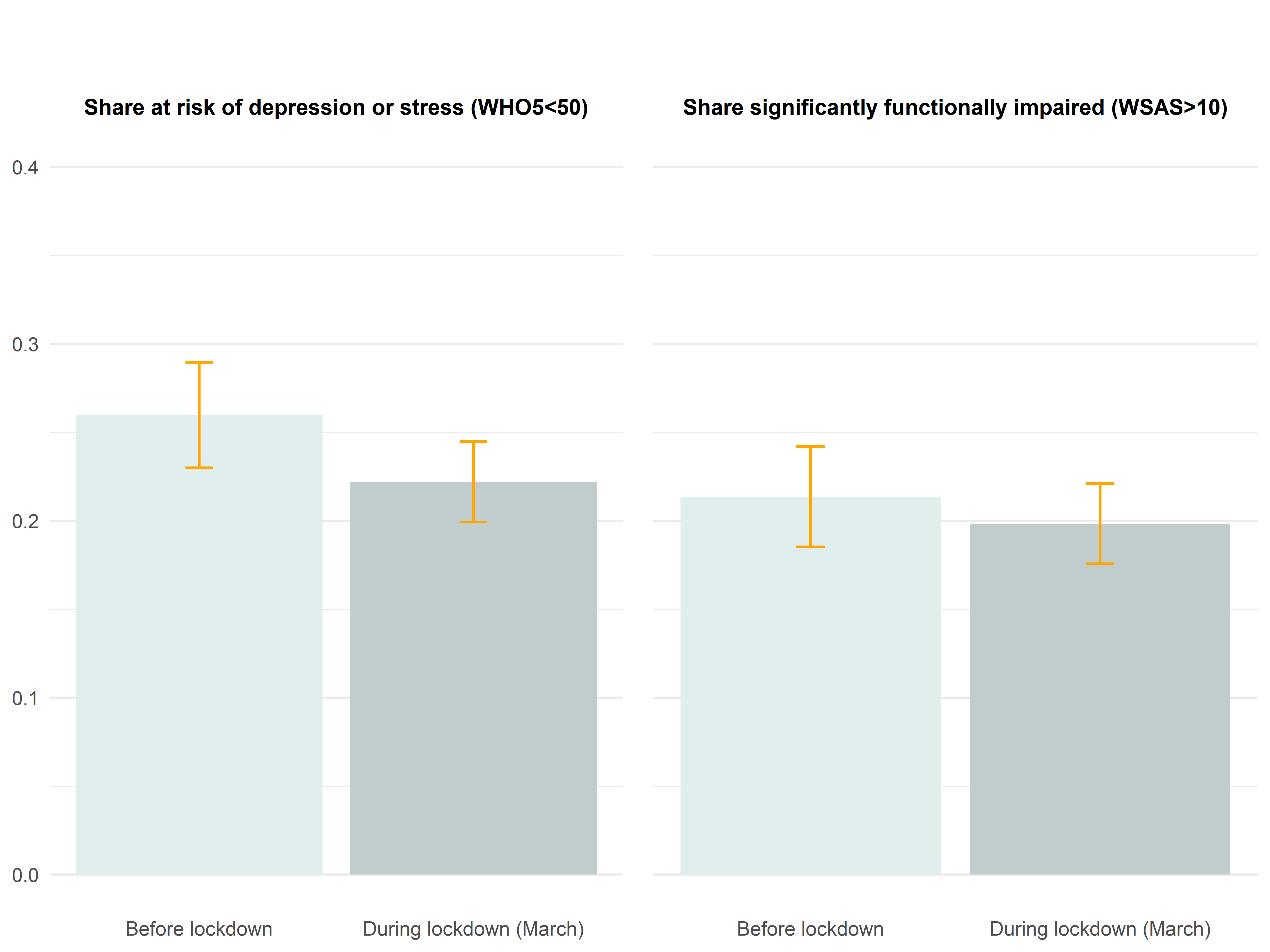


Figure A7. Proportion of respondents above WHO5 clinical threshold by household structure with re-weighting.


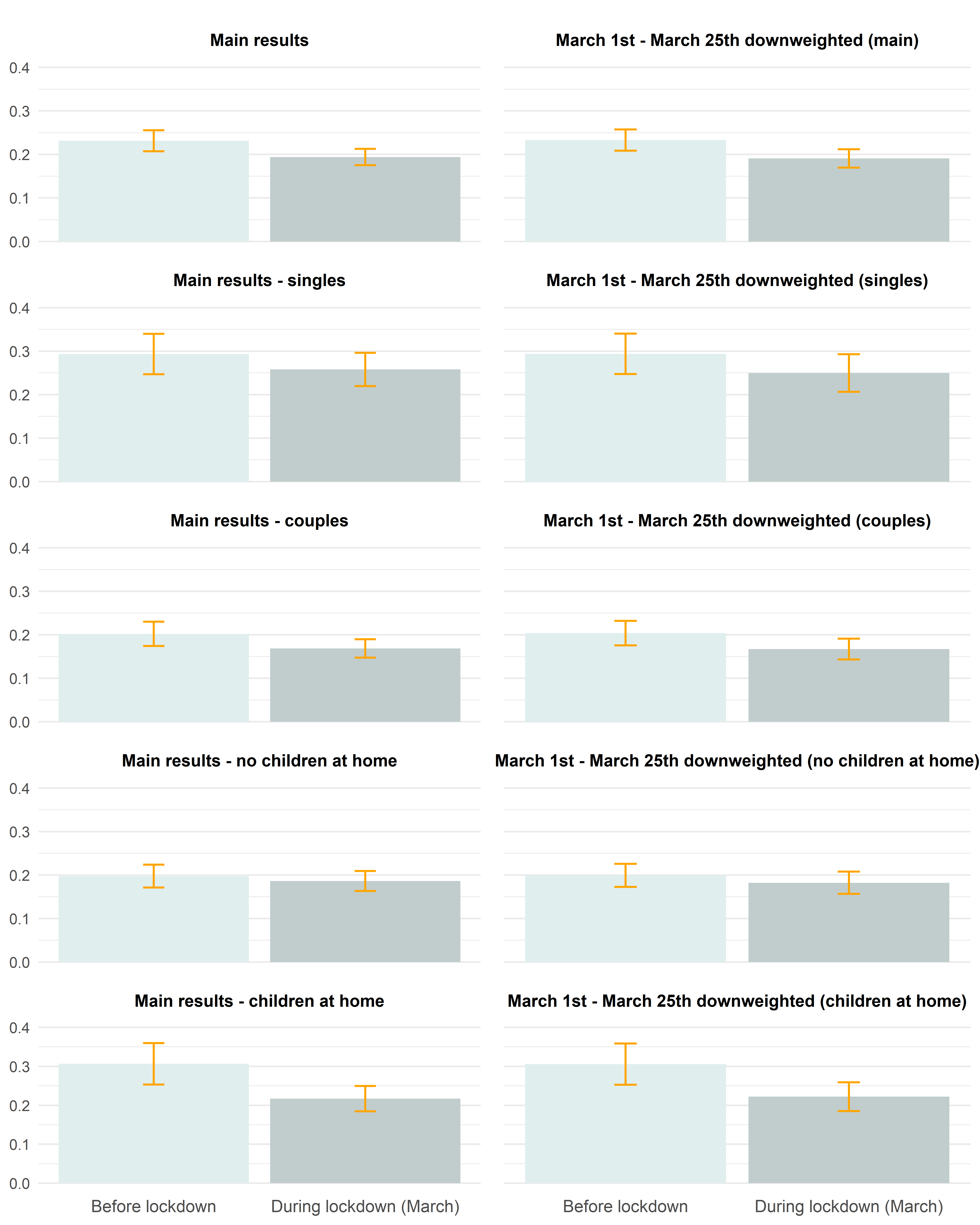


Note: The down weighting implies weighing individual responses by the numbers of days within the past two weeks prior to survey response that fell on or after the lockdown date, March 11.

Figure A8. Proportion of respondents above WSAS clinical threshold by household structure with re-weighting.


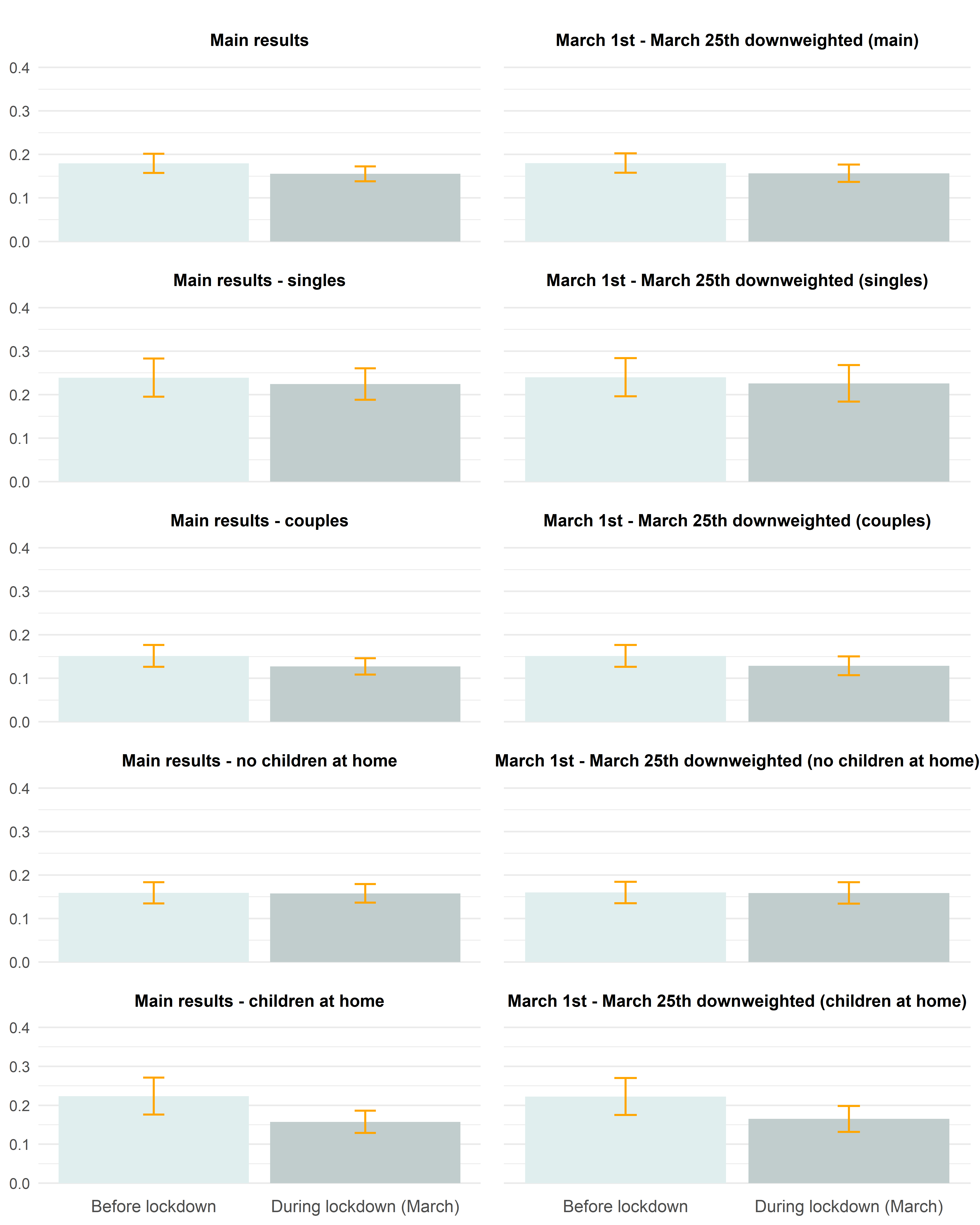


Note: The down weighting implies weighing individual responses by the numbers of days within the past two weeks prior to survey response that fell on or after the lockdown date, March 11.

Figure A9. Proportion of respondents above WHO5 clinical threshold for respondents with children by age of children.


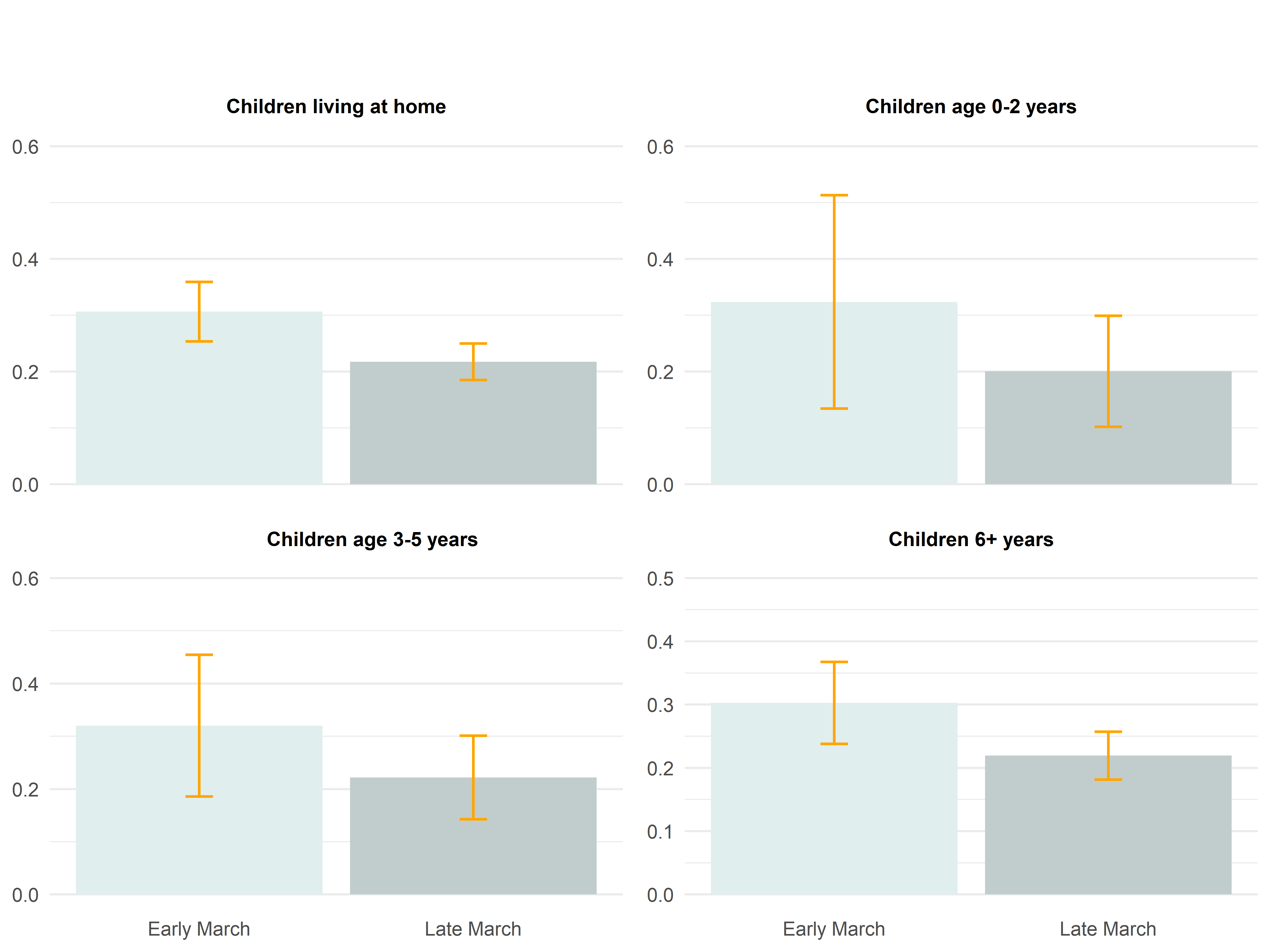


Figure A10. Proportion of respondents above WSAS clinical threshold for respondents with children by age of children.


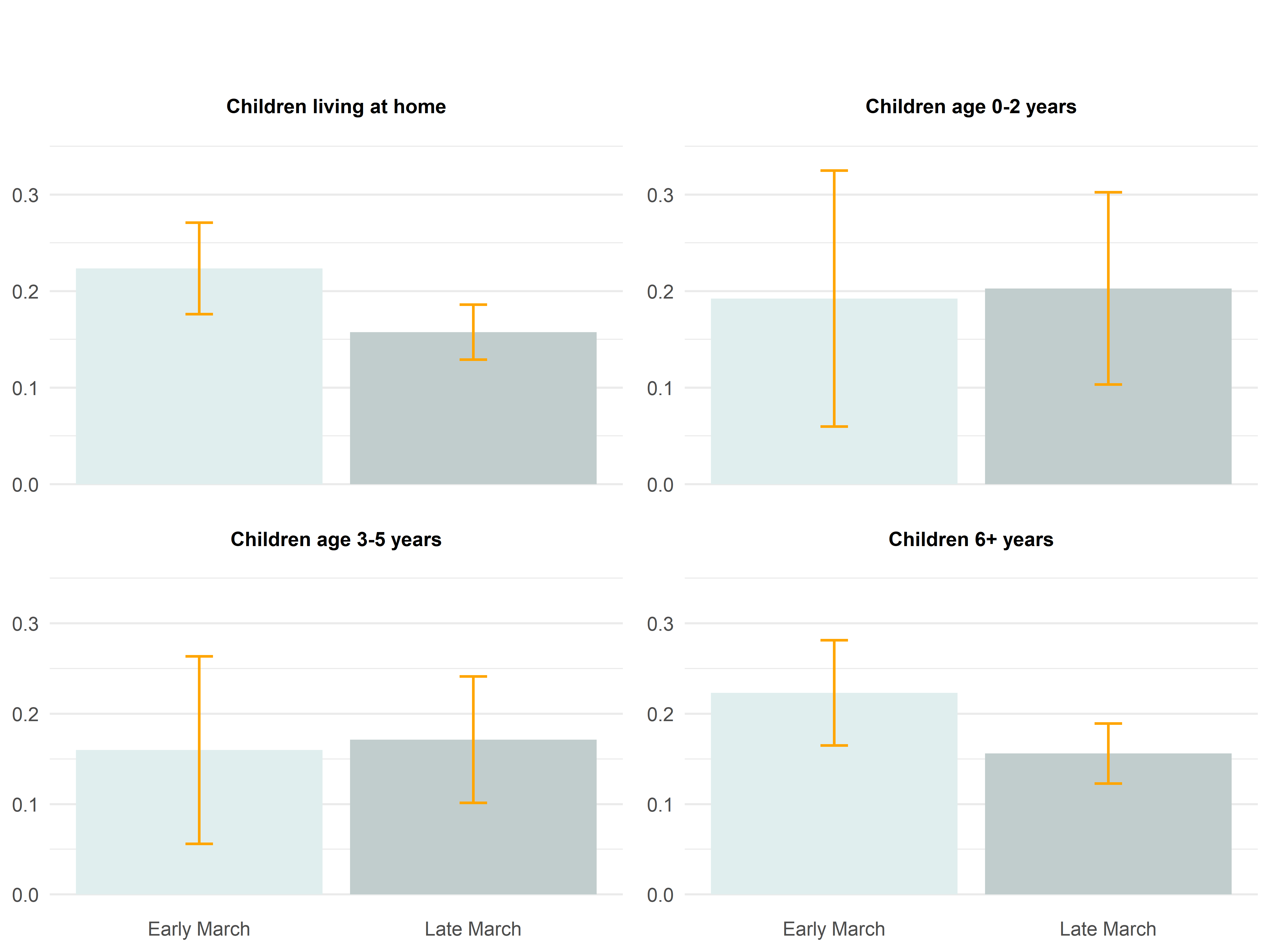


Table A1. Means of respondents’ background characteristics, by response date and by sample characteristic.

| Data type | Data from first survey wave | | |  | Panel data | | |
| --- | --- | --- | --- | --- | --- | --- | --- |
| Variable | Early  March | Late  March | p |  | Responded  Early March  and July | Responded  Early March  but not July | p |
| Male | 0.447 | 0.450 | 0.885 |  | 0.457 | 0.426 | 0.319 |
| Female | 0.553 | 0.550 | 0.885 |  | 0.543 | 0.574 | 0.319 |
| Ages 18-29 | 0.137 | 0.139 | 0.843 |  | 0.107 | 0.198 | 0.000 |
| Ages 30-39 | 0.089 | 0.122 | 0.005 |  | 0.080 | 0.107 | 0.134 |
| Ages 40-49 | 0.135 | 0.172 | 0.008 |  | 0.106 | 0.195 | <0.001 |
| Ages 50-59 | 0.208 | 0.238 | 0.062 |  | 0.212 | 0.198 | 0.575 |
| Ages 60-69 | 0.238 | 0.183 | <0.001 |  | 0.274 | 0.162 | <0.001 |
| Ages 70-79 | 0.194 | 0.146 | 0.001 |  | 0.220 | 0.140 | 0.001 |
| Singles | 0.312 | 0.294 | 0.307 |  | 0.304 | 0.330 | 0.386 |
| Couples | 0.688 | 0.706 | 0.307 |  | 0.696 | 0.670 | 0.386 |
| No children at home | 0.736 | 0.634 | <0.001 |  | 0.773 | 0.659 | <0.001 |
| Children at home | 0.264 | 0.366 | <0.001 |  | 0.227 | 0.341 | <0.001 |
| Northern Jutland | 0.103 | 0.104 | 0.917 |  | 0.102 | 0.104 | 0.911 |
| Central Jutland | 0.241 | 0.247 | 0.735 |  | 0.236 | 0.253 | 0.537 |
| Southern Denmark | 0.232 | 0.193 | 0.010 |  | 0.241 | 0.214 | 0.318 |
| Capitol | 0.274 | 0.308 | 0.051 |  | 0.265 | 0.294 | 0.304 |
| Zealand | 0.149 | 0.148 | 0.940 |  | 0.156 | 0.135 | 0.347 |
| In Job | 0.592 | 0.692 | <0.001 |  | 0.579 | 0.618 | 0.215 |
| Unemployed | 0.022 | 0.018 | 0.382 |  | 0.025 | 0.016 | 0.370 |
| Outside labor force | 0.383 | 0.286 | <0.001 |  | 0.394 | 0.360 | 0.264 |
| WHO-5 < 50 | 0.224 | 0.200 | 0.123 |  | 0.208 | 0.255 | 0.076 |
| WSAS > 10 | 0.174 | 0.159 | 0.300 |  | 0.151 | 0.223 | 0.003 |
| N | 1,127 | 1,709 |  |  | 763 | 364 |  |

Notes: Table shows means and p-values refer to T-tests of statistically detectable differences across table columns within data type.

Table A2. OLS regressions of being at risk of depression/stress (WHO5<50) and experiencing significant functional impairment (WSAS>10).

| Model | (1) | (2) | (3) | (4) |
| --- | --- | --- | --- | --- |
| Outcome | WHO5<50 | WHO5<50 | WSAS>10 | WSAS>10 |
| Before 3/11/20 | 0.291*** | 0.364*** | 0.183*** | 0.307*** |
|  | (0.026) | (0.038) | (0.022) | (0.034) |
| After 3/11/20 | -0.037* | -0.029 | -0.024 | -0.013 |
|  | (0.018) | (0.016) | (0.014) | (0.014) |
| Female | 0.024 | 0.014 | 0.022 | 0.010 |
|  | (0.015) | (0.015) | (0.014) | (0.014) |
| Ages 18-29 | -0.015 | -0.059 | 0.072* | 0.008 |
|  | (0.031) | (0.032) | (0.029) | (0.029) |
| Ages 30-39 | -0.008 | -0.014 | 0.054 | 0.046 |
|  | (0.033) | (0.033) | (0.030) | (0.029) |
| Ages 50-59 | -0.065* | -0.065* | -0.012 | -0.019 |
|  | (0.027) | (0.028) | (0.023) | (0.024) |
| Ages 60-69 | -0.121*** | -0.160*** | -0.077*** | -0.150*** |
|  | (0.026) | (0.031) | (0.023) | (0.027) |
| Ages 70-79 | -0.185*** | -0.275*** | -0.076** | -0.222*** |
|  | (0.026) | (0.033) | (0.023) | (0.032) |
| Single |  | 0.077*** |  | 0.070*** |
|  |  | (0.018) |  | (0.016) |
| Children |  | 0.006 |  | -0.011 |
| at home |  | (0.022) |  | (0.019) |
| In Job |  | -0.121*** |  | -0.181*** |
|  |  | (0.020) |  | (0.020) |
| Unemployed |  | -0.071 |  | -0.171*** |
|  |  | (0.062) |  | (0.052) |
| North Jutland |  | 0.006 |  | 0.046 |
|  |  | (0.027) |  | (0.024) |
| Central |  | -0.004 |  | 0.041* |
| Jutland |  | (0.020) |  | (0.018) |
| Southern |  | 0.042 |  | 0.055** |
| Denmark |  | (0.022) |  | (0.020) |
| Zealand |  | 0.029 |  | 0.030 |
|  |  | (0.024) |  | (0.021) |
| *N* | 2,836 | 2,836 | 2,836 | 2,836 |

Standard errors in parentheses.

* p < 0.05, ** p < 0.01, *** p < 0.001

Table A3. Parameter estimates from OLS regressions of being at risk of depression/stress (WHO5<50), by household structure.

| Model | (1) | (2) | (3) | (4) |
| --- | --- | --- | --- | --- |
| Household structure | Singles | Couples | No children at home | Children at home |
| Before 3/11/20 | 0.435*** | 0.239*** | 0.355*** | 0.273*** |
|  | (0.053) | (0.029) | (0.045) | (0.039) |
| After 3/11/20 | -0.035 | -0.034 | -0.011 | -0.089** |
|  | (0.031) | (0.018) | (0.018) | (0.032) |
| Female | -0.031 | 0.045** | 0.009 | 0.068* |
|  | (0.031) | (0.017) | (0.018) | (0.029) |
| Ages 18-29 | -0.092 | -0.014 | -0.068 | -0.018 |
|  | (0.057) | (0.038) | (0.052) | (0.043) |
| Ages 30-39 | -0.028 | -0.011 | -0.043 | -0.009 |
|  | (0.069) | (0.037) | (0.060) | (0.039) |
| Ages 50-59 | -0.137* | -0.040 | -0.155** | -0.005 |
|  | (0.057) | (0.030) | (0.048) | (0.037) |
| Ages 60-69 | -0.192*** | -0.096** | -0.193*** | -0.002 |
|  | (0.056) | (0.029) | (0.046) | (0.090) |
| Ages 70-79 | -0.237*** | -0.166*** | -0.255*** | -0.184*** |
|  | (0.057) | (0.028) | (0.046) | (0.029) |
| *N* | 855 | 1981 | 1,913 | 923 |

Standard errors in parentheses

* *p* < 0.05, ** *p* < 0.01, *** *p* < 0.001

Table A4. OLS regressions of being at risk of significant functional impairment (WSAS>10), by household structure.

| Model | (1) | (2) | (3) | (4) |
| --- | --- | --- | --- | --- |
| Household structure | Singles | Couples | No children at home | Children at home |
| Before 3/11/20 | 0.331*** | 0.131*** | 0.267*** | 0.150*** |
|  | (0.051) | (0.023) | (0.042) | (0.033) |
| After 3/11/20 | -0.015 | -0.024 | -0.001 | -0.066* |
|  | (0.029) | (0.016) | (0.017) | (0.028) |
| Female | -0.029 | 0.040** | 0.009 | 0.066** |
|  | (0.029) | (0.015) | (0.017) | (0.025) |
| Ages 18-29 | -0.048 | 0.106** | -0.010 | 0.091* |
|  | (0.054) | (0.036) | (0.049) | (0.041) |
| Ages 30-39 | 0.027 | 0.053 | 0.021 | 0.046 |
|  | (0.067) | (0.033) | (0.058) | (0.035) |
| Ages 50-59 | -0.077 | 0.009 | -0.111* | 0.039 |
|  | (0.054) | (0.025) | (0.044) | (0.031) |
| Ages 60-69 | -0.146** | -0.054* | -0.170*** | 0.059 |
|  | (0.053) | (0.023) | (0.042) | (0.083) |
| Ages 70-79 | -0.161** | -0.046 | -0.166*** | -0.084*** |
|  | (0.054) | (0.025) | (0.043) | (0.024) |
| *N* | 855 | 1,981 | 1,913 | 923 |

Standard errors in parentheses

* *p* < 0.05, ** *p* < 0.01, *** *p* < 0.001

Table A5. OLS regressions of WHO5<50 and WSAS>10 for repeat responses in early March and July.

| Model | (1) | (2) | (3) | (4) |
| --- | --- | --- | --- | --- |
| Outcome | WHO5<50 | WHO5<50 | WSAS>10 | WSAS>10 |
| Early March | 0.294*** | 0.332*** | 0.166*** | 0.248*** |
|  | (0.047) | (0.067) | (0.046) | (0.066) |
| July | -0.033* | -0.033* | 0.199*** | 0.199*** |
|  | (0.014) | (0.014) | (0.017) | (0.017) |
| Female | 0.011 | 0.006 | 0.000 | -0.008 |
|  | (0.025) | (0.025) | (0.026) | (0.026) |
| Ages 18-29 | -0.009 | -0.034 | 0.064 | 0.027 |
|  | (0.058) | (0.058) | (0.062) | (0.063) |
| Ages 30-39 | 0.027 | 0.026 | 0.054 | 0.052 |
|  | (0.070) | (0.068) | (0.066) | (0.063) |
| Ages 50-59 | -0.095 | -0.094 | -0.028 | -0.023 |
|  | (0.051) | (0.053) | (0.052) | (0.051) |
| Ages 60-69 | -0.123* | -0.150* | -0.043 | -0.083 |
|  | (0.050) | (0.058) | (0.049) | (0.056) |
| Ages 70-79 | -0.175*** | -0.222*** | -0.039 | -0.115 |
|  | (0.049) | (0.060) | (0.049) | (0.060) |
| Single |  | 0.059* |  | 0.048 |
|  |  | (0.029) |  | (0.028) |
| Children |  | -0.005 |  | 0.005 |
| at home |  | (0.040) |  | (0.040) |
| In Job |  | -0.064* |  | -0.116** |
|  |  | (0.032) |  | (0.035) |
| Unemployed |  | 0.029 |  | -0.039 |
|  |  | (0.094) |  | (0.090) |
| North Jutland |  | -0.019 |  | 0.004 |
|  |  | (0.043) |  | (0.047) |
| Central |  | -0.004 |  | -0.004 |
| Jutland |  | (0.034) |  | (0.036) |
| Southern |  | 0.001 |  | 0.017 |
| Denmark |  | (0.033) |  | (0.036) |
| Zealand |  | 0.043 |  | 0.007 |
|  |  | (0.041) |  | (0.040) |
| *N* | 1,526 | 1,526 | 1,526 | 1,526 |

Standard errors in parentheses and clustered at the individual level.

* *p* < 0.05, ** *p* < 0.01, *** *p* < 0.001

Table A6. OLS regressions of being at risk of depression/stress (WHO5<50), by household structure.

| Model | (1) | (2) | (3) | (4) |
| --- | --- | --- | --- | --- |
| Household structure | Singles | Couples | No children at home | Children at home |
| Early March | 0.440*** | 0.284*** | 0.374*** | 0.322*** |
|  | (0.075) | (0.044) | (0.064) | (0.057) |
| July | -0.047 | -0.033* | -0.006 | -0.133*** |
|  | (0.027) | (0.016) | (0.015) | (0.032) |
| Female | -0.042 | 0.026 | -0.000 | 0.020 |
|  | (0.044) | (0.025) | (0.025) | (0.047) |
| Ages 18-29 | -0.061 | -0.048 | -0.053 | -0.033 |
|  | (0.083) | (0.057) | (0.076) | (0.061) |
| Ages 30-39 | -0.014 | -0.021 | -0.072 | 0.022 |
|  | (0.108) | (0.065) | (0.093) | (0.071) |
| Ages 50-59 | -0.170* | -0.109* | -0.177* | -0.104 |
|  | (0.084) | (0.049) | (0.069) | (0.059) |
| Ages 60-69 | -0.167* | -0.137** | -0.211** | 0.266 |
|  | (0.083) | (0.047) | (0.066) | (0.222) |
| Ages 70-79 | -0.271*** | -0.189*** | -0.274*** | NA |
|  | (0.078) | (0.046) | (0.065) |  |
| *N* | 584 | 1,306 | 1,420 | 470 |

Standard errors in parentheses and clustered at the individual level. NA = Not Available.

* *p* < 0.05, ** *p* < 0.01, *** *p* < 0.001
Note: Repeat responses in early March and July.

Table A7. Parameter estimates from OLS regressions of WSAS > 10, by household structure.

| Model | (1) | (2) | (3) | (4) |
| --- | --- | --- | --- | --- |
| Household structure | Singles | Couples | No children at home | Children at home |
| Early March | 0.388*** | 0.117** | 0.228*** | 0.163** |
|  | (0.075) | (0.040) | (0.061) | (0.050) |
| July | 0.162*** | 0.191*** | 0.214*** | 0.089* |
|  | (0.033) | (0.020) | (0.020) | (0.035) |
| Female | -0.110* | 0.061* | -0.016 | 0.087 |
|  | (0.043) | (0.026) | (0.025) | (0.048) |
| Ages 18-29 | -0.051 | 0.090 | 0.055 | 0.052 |
|  | (0.085) | (0.063) | (0.074) | (0.072) |
| Ages 30-39 | 0.032 | -0.007 | 0.057 | -0.029 |
|  | (0.102) | (0.059) | (0.087) | (0.066) |
| Ages 50-59 | -0.128 | -0.010 | -0.092 | 0.007 |
|  | (0.085) | (0.047) | (0.066) | (0.061) |
| Ages 60-69 | -0.128 | -0.034 | -0.099 | 0.203 |
|  | (0.083) | (0.044) | (0.063) | (0.173) |
| Ages 70-79 | -0.186* | -0.007 | -0.102 | NA |
|  | (0.078) | (0.046) | (0.063) |  |
| *N* | 584 | 1,306 | 1,420 | 470 |

Standard errors in parentheses and clustered at the individual level. NA = Not Available.

* *p* < 0.05, ** *p* < 0.01, *** *p* < 0.001
Notes: Repeat responses in early March and July.

Table A8. Summary of robustness check of results’ sensitivity to cutoffs on the WHO5 and WSAS scales.

|  | (1) | (2) | (3) | (4) | (5) | (6) |
| --- | --- | --- | --- | --- | --- | --- |
|  | WHO5 raw  score < 13 | WHO5  < 40 | WHO5  < 45 | WHO5  < 50 | WHO5  < 55 | WHO5  < 60 |
| Before 3/11/20 | 0.291*** | 0.183*** | 0.249*** | 0.291*** | 0.327*** | 0.372*** |
|  | (0.026) | (0.023) | (0.025) | (0.026) | (0.027) | (0.028) |
| After 3/11/20 | -0.037* | -0.032* | -0.030* | -0.037* | -0.032 | -0.030 |
|  | (0.016) | (0.013) | (0.015) | (0.016) | (0.017) | (0.017) |
| *N* | 2,836 | 2,836 | 2,836 | 2,836 | 2,836 | 2,836 |
|  | (7) | (8) | (9) | (10) | (11) | (12) |
|  | WSAS  > 5 | WSAS  > 8 | WSAS  > 10 | WSAS  > 12 | WSAS  > 15 | WSAS  > 20 |
| Before 3/11/20 | 0.304*** | 0.229*** | 0.183*** | 0.161*** | 0.128*** | 0.062*** |
|  | (0.027) | (0.024) | (0.022) | (0.021) | (0.019) | (0.014) |
| After 3/11/20 | -0.014 | -0.024 | -0.024 | -0.032* | -0.025* | -0.003 |
|  | (0.017) | (0.015) | (0.014) | (0.013) | (0.012) | (0.009) |
| *N* | 2,836 | 2,836 | 2,836 | 2,836 | 2,836 | 2,836 |

Standard errors in parentheses. All models control for gender and age.

WHO5<50 signals increased risk of depression and/or stress.

WHO5 raw score <13 signals very poor wellbeing.

WSAS>10 signals significant functional impairment.

WSAS>20 signals moderately severe or worse psychopathology.

* *p* < 0.05, ** *p* < 0.01, *** *p* < 0.001

Table A9. Summary of results from individual level fixed effects specification

|  | (1) | (2) | (3) | (4) | (5) |
| --- | --- | --- | --- | --- | --- |
| WHO-5 < 50 | All | Singles | Couples | No Children at home | Children at home |
| Before 3/11/20 | 0.212^***^ | 0.275^***^ | 0.185^***^ | 0.177^***^ | 0.324^***^ |
|  | (0.012) | (0.022) | (0.014) | (0.012) | (0.029) |
| After 3/11/20 | -0.016 | -0.022 | -0.014 | 0.016 | -0.128^**^ |
|  | (0.020) | (0.038) | (0.023) | (0.022) | (0.043) |
| Time trend | -0.033^*^ | -0.035 | -0.032^*^ | 0.000 | -0.145^***^ |
| up to July | (0.014) | (0.026) | (0.016) | (0.014) | (0.034) |
| *NxT* | 4,393 | 1,323 | 3,070 | 3,054 | 1,339 |
|  | (6) | (7) | (8) | (9) | (10) |
| WSAS > 10 | All | Singles | Couples | No Children at home | Children at home |
| Before 3/11/20 | 0.157^***^ | 0.231^***^ | 0.126^***^ | 0.148^***^ | 0.204^***^ |
|  | (0.014) | (0.026) | (0.017) | (0.016) | (0.029) |
| After 3/11/20 | -0.002 | -0.026 | 0.008 | -0.002 | -0.041 |
|  | (0.023) | (0.043) | (0.028) | (0.028) | (0.042) |
| Time trend | 0.199^***^ | 0.190^***^ | 0.203^***^ | 0.232^***^ | 0.087^*^ |
| up to July | (0.017) | (0.032) | (0.020) | (0.020) | (0.034) |
| *NxT* | 4,393 | 1,323 | 3,070 | 3,054 | 1,339 |

Standard errors in parentheses

* *p* < 0.05, ** *p* < 0.01, *** *p* < 0.001
