# Supplementary material for: Risk of stress/depression and functional impairment in Denmark immediately following a COVID-19 shutdown
Source: BMC Public Health. 2021 May 26;21:984. doi: 10.1186/s12889-021-11020-3 (PMC8149922; doi:10.1186/s12889-021-11020-3)
Supplement: Supplementary file 2 — Additional file 2. [file 12889_2021_11020_MOESM2_ESM.pdf]

```
///////////////////////////////////////////////////////////////////////////////
//
//  Title: RISK OF STRESS/DEPRESSION AND FUNCTIONAL IMPAIRMENT IN DENMARK
//         IMMEDIATELY FOLLOWING A COVID-19 SHUTDOWN
//
//  Authors: Andersen, Fallesen, and Bruckner
//
//  This do-file compiles data from survey data files and prints all results
//
///////////////////////////////////////////////////////////////////////////////

version 15
set more off
set seed 61666738
cd y:/data/workdata/707676/temp/    // <-- Make sure to change working directory


///////////////////////////////////////////////////////////////////////////////
//  Check coding of survey data
///////////////////////////////////////////////////////////////////////////////

  * check coding in March data
  use y:/data/workdata/707676/LHA_PF/data/regionhpsykiatri2020_svar.dta, clear
  des
  destring pnr, replace
  sum pnr koen aldergrp region famtype HjembBoern ///sociodemographics
      pervgt pervgt_1 pervgt_2      /// statistical weights from Stat. Denmark
      F1 F2 F3 F4 F5                /// WHO-5 items (5)
      C1 C2 C3 C4 C5                // WSAS items (5)

  * keep only relevant variables for our analyses
  ge time1=int_dato<mdy(3,11,2020)
  ge time2=int_dato>=mdy(3,11,2020)
  ge time=1 if time1==1
  replace time=2 if time2==1
  keep pnr int_dato time time1 time2 koen aldergrp region famtype HjembBoern ///
      pervgt F1 F2 F3 F4 F5 C1 C2 C3 C4 C5
  fre koen aldergrp region famtype HjembBoern F1 F2 F3 F4 F5 C1 C2 C3 C4 C5
  * coding looks correct, save March data
  save data_march.dta, replace

  * save sociodemographics from wave 1 to merge onto wave 2 data
  keep pnr koen aldergrp region famtype HjembBoern
  save data_background.dta, replace


  * check coding in July data
  use y:/data/workdata/707676/LHA_PF/data/rffpsykiatri2020_m1_svar.dta, clear
   destring pnr, replace
  merge 1:1 pnr using data_background.dta // looks good, all in July found in March
  keep if _merge==3 // keeping only balanced panel
  drop _merge
  des
  rename (fam Inter) (Fam Intern)   // correcting inconsistent variable names
  sum pnr koen aldergrp region famtype HjembBoern ///sociodemographics
      F1 F2 F3 F4 F5                /// WHO-5 items (5)
      C1 C2 C3 C4 C5                // WSAS items (5)
  ge time3=1
  ge time=3

  keep pnr int_dato time time3 koen aldergrp region famtype HjembBoern ///
      F1 F2 F3 F4 F5 C1 C2 C3 C4 C5
  fre koen aldergrp region famtype HjembBoern F1 F2 F3 F4 F5 C1 C2 C3 C4 C5
  * coding looks correct, save July data
  save data_july.dta, replace


///////////////////////////////////////////////////////////////////////////////
//  Check scale reliability using Cronbach's alpha
///////////////////////////////////////////////////////////////////////////////

  * Check scales in March data
  use data_march.dta, clear
  * WHO-5
  alpha F1 F2 F3 F4 F5  // alpha = 0.9145
```

```
  * WSAS
  alpha C1 C2 C3 C4 C5  // alpha = 0.9028

  * Check scales in July data
  use data_july.dta, clear
  * WHO-5
  alpha F1 F2 F3 F4 F5  // alpha = 0.9109
  * WSAS
  alpha C1 C2 C3 C4 C5  // alpha = 0.8941


////////////////////////////////////////////////////////////////////////////////
//  Calculate WHO-5 and WSAS scales
////////////////////////////////////////////////////////////////////////////////

  * Calculate scales on March data
  use data_march.dta, clear

  * WHO-5: raw sum multplied by 4
  fre F1 F2 F3 F4 F5        // missing is already set to .
  ge WHO5=(F1+F2+F3+F4+F5)*4
  tab WHO5

  * WHO-5 <=50: increased risk of depression and/or stress
  ge WHO5_50=WHO5 <=50 & WHO5!=.

  * WHO5 raw score <13:  very poor wellbeing
  ge WHO5_13=(F1+F2+F3+F4+F5)<13 & WHO5!=.

  * WSAS: raw sum of the five items
  fre C1 C2 C3 C4 C5        // missing is already set to .
  ge WSAS=C1+C2+C3+C4+C5
  tab WSAS

  * WSAS > 10: significant functional impairment
  ge WSAS_10=WSAS>10 & WSAS!=.

  * Save March data
  save data_march_scales.dta, replace


  * Calculate scales on July data
  use data_july.dta, clear

  * WHO-5: raw sum multplied by 4
  fre F1 F2 F3 F4 F5        // missing is already set to .
  ge WHO5=(F1+F2+F3+F4+F5)*4
  tab WHO5

  * WHO-5 <=50: increased risk of depression and/or stress
  ge WHO5_50=WHO5 <=50 & WHO5!=.

  * WHO5 raw score <13:  very poor wellbeing
  ge WHO5_13=(F1+F2+F3+F4+F5)<13 & WHO5!=.

  * WSAS: raw sum of the five items
  fre C1 C2 C3 C4 C5        // missing is already set to .
  ge WSAS=C1+C2+C3+C4+C5
  tab WSAS

  * WSAS > 10: significant functional impairment
  ge WSAS_10=WSAS>10 & WSAS!=.

  * Save March data
  save data_july_scales.dta, replace


////////////////////////////////////////////////////////////////////////////////
//  Create unbalanced and balanced panels,
//  add controls from registers, and recode
////////////////////////////////////////////////////////////////////////////////

  * Balanced panel (i.e responded both March and July)
  use data_march_scales.dta, clear
  append using data_july_scales.dta
```

```
  egen g=rowmiss(pnr WHO5 WSAS)
  bysort pnr: egen h=max(g)
  drop if g>0
  drop h g

  forval f=1/3{
        replace time`f'=0 if time`f'==.
  }
  bysort pnr: keep if _N==2

  * code sociodemographics
  ge Male=koen==1
  ge Female=koen==2

  ge Age1829=aldergrp==1
  ge Age3039=aldergrp==2
  ge Age4049=aldergrp==3
  ge Age5059=aldergrp==4
  ge Age6069=aldergrp==5
  ge Age7079=aldergrp==6

  ge NorthernJutland=region==81
  ge CentralJutland=region==82
  ge SouthernJutland=region==83
  ge Capitol=region==84
  ge Zealand=region==85

  ge Single=inlist(famtype,1,2)
  ge Couple=inlist(famtype,3,4)

  ge Children=HjembBoern==1
  ge NoChildren=HjembBoern==2

  * create family characteristics
  preserve
    use familie_id alder using e:/data/rawdata/707676/bef2019.dta, clear
    ge I=inrange(alder,0,1)     // count as infants anyone born in 2019
                                // (1 yr in 2020) and up to 2 years on
                                // Jan. 1st 2020
    ge T=inrange(alder,2,4)     // count as toddlers anyone ages 3 to 5 on
                                // Jan. 1st 2020
    ge S=inrange(alder,5,18)    // count as school age children anyone ages
                                // 6 to 19 on Jan 1st 2020
    bysort familie_id: egen Infants=sum(I)
    bysort familie_id: egen Toddlers=sum(T)
    bysort familie_id: egen Schoolers=sum(S)
    bysort familie_id: keep if _n==1
    drop if familie_id==.
    keep familie_id Infants Toddlers Schoolers
    save fam_char.dta, replace
  restore

  merge m:1 pnr using e:/data/rawdata/707676/bef2019.dta, nogen keep(1 3) keepus(familie_id)
  merge m:1 familie_id using fam_char.dta, nogen keep(1 3)

  * top code number of children
  replace Infants=3 if Infants>3
  replace Toddlers=3 if Toddlers>3
  replace Schoolers=3 if Schoolers>3

  * add socioeconomic status
  preserve
    bysort pnr (int_dato): keep if _n==1
    merge 1:m pnr using e:/data/rawdata/707676/ras2018.dta, nogen keep(1 3) keepus(soc* prim*)
    replace prim=99 if prim==0
    bysort pnr (primaer_status_kode): keep if _n==1
    ta prim
    ge InJob=inrange(soc_status_kode,110,138)
    ge Unemployed=soc_status_kode==200
    ge OLF=inrange(soc_status_kode,300,612)
    keep pnr InJob Unemployed OLF
    save ras.dta, replace
```

```
  restore
  merge m:1 pnr using ras.dta, nogen keep(1 3)

  * tidy up data
  keep pnr int_dato time time1 time2 time3 ///
       koen aldergrp region famtype HjembBoern ///
       pervgt F1 F2 F3 F4 F5 C1 C2 C3 C4 C5 ///
       WHO5 WHO5_50 WHO5_13 WSAS WSAS_10 ///
       Male Female Age* ///
       NorthernJutland CentralJutland SouthernJutland Capitol Zealand ///
       Single Couple Children NoChildren Infants Toddlers Schoolers ///
       InJob Unemp OLF

  order pnr int_dato time time1 time2 time3 ///
       koen aldergrp region famtype HjembBoern ///
       pervgt F1 F2 F3 F4 F5 C1 C2 C3 C4 C5 ///
       WHO5 WHO5_50 WHO5_13 WSAS WSAS_10 ///
       Male Female Age* ///
       NorthernJutland CentralJutland SouthernJutland Capitol Zealand ///
       Single Couple Children NoChildren Infants Toddlers Schoolers ///
       InJob Unemp OLF

  * save
  save panel_balanced_march_july.dta, replace


  * Unbalaned panel (i.e. all respondents in March + respondents in June)
  use data_march_scales.dta, clear
  append using data_july_scales.dta

  * code background
  ge Male=koen==1
  ge Female=koen==2

  ge Age1829=aldergrp==1
  ge Age3039=aldergrp==2
  ge Age4049=aldergrp==3
  ge Age5059=aldergrp==4
  ge Age6069=aldergrp==5
  ge Age7079=aldergrp==6

  ge NorthernJutland=region==81
  ge CentralJutland=region==82
  ge SouthernJutland=region==83
  ge Capitol=region==84
  ge Zealand=region==85

  ge Single=inlist(famtype,1,2)
  ge Couple=inlist(famtype,3,4)

  ge Children=HjembBoern==1
  ge NoChildren=HjembBoern==2

 * create family characteristics
  preserve
    use familie_id alder using e:/data/rawdata/707676/bef2019.dta, clear
    ge I=inrange(alder,0,1)      // count as infants anyone born in 2019
                                 // (1 yr in 2020) and up to 2 years on
                                 // Jan. 1st 2020
    ge T=inrange(alder,2,4)      // count as toddlers anyone ages 3 to 5 on
                                 // Jan. 1st 2020
    ge S=inrange(alder,5,18)     // count as school age children anyone ages
                                 // 6 to 19 on Jan 1st 2020
    bysort familie_id: egen Infants=sum(I)
    bysort familie_id: egen Toddlers=sum(T)
    bysort familie_id: egen Schoolers=sum(S)
    bysort familie_id: keep if _n==1
    drop if familie_id==.
    keep familie_id Infants Toddlers Schoolers
    save fam_char.dta, replace
  restore

  merge m:1 pnr using e:/data/rawdata/707676/bef2019.dta, nogen keep(1 3) keepus(familie_id)
  merge m:1 familie_id using fam_char.dta, nogen keep(1 3)
```

```

  * top code number of children
  replace Infants=3 if Infants>3
  replace Toddlers=3 if Toddlers>3
  replace Schoolers=3 if Schoolers>3

  * add socioeconomic status
  preserve
    bysort pnr (int_dato): keep if _n==1
    merge 1:m pnr using e:/data/rawdata/707676/ras2018.dta, nogen keep(1 3) keepus(soc* prim*)
    replace prim=99 if prim==0
    bysort pnr (primaer_status_kode): keep if _n==1
    ta prim
    ge InJob=inrange(soc_status_kode,110,138)
    ge Unemployed=soc_status_kode==200
    ge OLF=inrange(soc_status_kode,300,612)
    keep pnr InJob Unemployed OLF
    save ras.dta, replace
  restore
  merge m:1 pnr using ras.dta, nogen keep(1 3)

  * fix time dummies
  forval f=1/3{
    replace time`f'=0 if time`f'==.
  }

  * tidy up data
  keep pnr int_dato time time1 time2 time3 ///
       koen aldergrp region famtype HjembBoern ///
       pervgt F1 F2 F3 F4 F5 C1 C2 C3 C4 C5 ///
       WHO5 WHO5_50 WHO5_13 WSAS WSAS_10 ///
       Male Female Age* ///
       NorthernJutland CentralJutland SouthernJutland Capitol Zealand ///
       Single Couple Children NoChildren Infants Toddlers Schoolers ///
       InJob Unemp OLF

  order pnr int_dato time time1 time2 time3 ///
       koen aldergrp region famtype HjembBoern ///
       pervgt F1 F2 F3 F4 F5 C1 C2 C3 C4 C5 ///
       WHO5 WHO5_50 WHO5_13 WSAS WSAS_10 ///
       Male Female Age* ///
       NorthernJutland CentralJutland SouthernJutland Capitol Zealand ///
       Single Couple Children NoChildren Infants Toddlers Schoolers ///
       InJob Unemp OLF

  * save
  save panel_unbalanced_march_july.dta, replace


////////////////////////////////////////////////////////////////////////////////
//  Table A1: Mean of respondents' background characteristics, by response
//            date and by sample characteristic
////////////////////////////////////////////////////////////////////////////////

  * First two table columns: Out-selection from pre to post March 11 wave 1
  use panel_unbalanced_march_july.dta if WHO5!=. & WSAS!=., clear
  drop if time==3

  * mark post March 11 respondents
  ge d=int_dato>=mdy(3,11,2020)
  ta d

  * T-test covariate distribution across balance
  local n=0
  foreach v in Male Female ///
             Age1829 Age3039 Age4049 Age5059 Age6069 Age7079 ///
             Single Couple ///
             NoChildren Children ///
             Infants Toddlers Schoolers ///
             NorthernJutland CentralJutland SouthernJutland Capitol Zealand ///
             InJob Unemployed OLF ///
             WHO5_50 WSAS_10{
    preserve
        ttest `v', by(d)
```

```
            ge Variable="`v'"
            ge LateMarch=r(mu_2)
            ge EarlyMarch=r(mu_1)
            ge t=r(t)
            ge p=r(p)
            keep if _n==1
            keep Variable EarlyMarch LateMarch t p
            order Variable EarlyMarch LateMarch p
            local n=`n'+1
            ge n=`n'
            if `n'>1 append using Ttests1.dta
            sort n
            save Ttests1.dta, replace
        restore
    }
    use Ttests1.dta, clear
    export excel using TabelA1_1.xlsx, firstrow(variables) replace


    * Last two table columns: Check out-selection from March to July among
    *                          Early March respondents only

    use panel_unbalanced_march_july.dta if WSAS!=. & WHO5!=., clear
    ge balancedpanel=0
    merge 1:1 pnr time using panel_balanced_march_july.dta, keep(1 3) keepus(pnr)
    replace balancedpanel=1 if _merge==3
    bysort pnr: egen x=max(time2)
    drop if x==1
    bysort pnr (int_dato): keep if _n==1 // keeping only one obs per panelist
    ta balancedpanel

    * T-test covariate distribution across balance
    local n=0
    foreach v in Male Female ///
               Age1829 Age3039 Age4049 Age5059 Age6069 Age7079 ///
               Single Couple ///
               NoChildren Children ///
               Infants Toddlers Schoolers ///
               NorthernJutland CentralJutland SouthernJutland Capitol Zealand ///
               InJob Unemployed OLF ///
               WHO5_50 WSAS_10{
        preserve
            ttest `v', by(balancedpanel)
            ge Variable="`v'"
            ge In_panel=r(mu_2)
            ge Dropout=r(mu_1)
            ge t=r(t)
            ge p=r(p)
            keep if _n==1
            keep Variable In_panel Dropout t p
            order Variable In_panel Dropout p
            local n=`n'+1
            ge n=`n'
            if `n'>1 append using Ttests2.dta
            sort n
            save Ttests2.dta, replace
        restore
    }
    use Ttests.dta, clear
    export excel using TableA1_2.xlsx, firstrow(variables) replace


  ////////////////////////////////////////////////////////////////////////////////
  //  Table 1: Descriptive statistis of sociodemographic characteristics of
  //           survey participants and the population of 18-79-year-old people
  //           in Denmark
  ////////////////////////////////////////////////////////////////////////////////

    * First, response rates reported in first subsection of Results
    use panel_unbalanced_march_july.dta if WHO5!=. & WSAS!=., clear
    ge wave=2
    replace wave=1 if int_dato<mdy(5,15,2020)
    ta wave
    ge balancedpanel=0
    merge 1:1 pnr time using panel_balanced_march_july.dta, keep(1 3) keepus(pnr)
```

```
    replace balancedpanel=1 if _merge==3
    ta balancedpanel

    ta wave balancedpanel
    table wave, c(mean balancedpanel)
    // 55 % of 1. wave participants answered 2. wave

    ge lockdown=int_dato>=mdy(3,11,2020)
    ta lockdown if wave==1
    // 40% pre lockdown, 60% post lockdown

    * Producing population for Table 1
    use Y:\Data\Workdata\707676\LHA_PF\data\rffpsykiatri2020_m1_pop.dta, clear
    destring pnr, force replace
    set seed 61666378

    * create family characteristics
    preserve
      use familie_id alder using e:/data/rawdata/707676/bef2019.dta, clear
      ge I=inrange(alder,0,1)      // count as infants anyone born in 2019
                                   // (1 yr in 2020) and up to 2 years on
                                   // Jan. 1st 2020
      ge T=inrange(alder,2,4)      // count as toddlers anyone ages 3 to 5 on
                                   // Jan. 1st 2020
      ge S=inrange(alder,5,18)     // count as school age children anyone ages
                                   // 6 to 19 on Jan 1st 2020
      bysort familie_id: egen Infants=sum(I)
      bysort familie_id: egen Toddlers=sum(T)
      bysort familie_id: egen Schoolers=sum(S)
      bysort familie_id: keep if _n==1
      drop if familie_id==.
      keep familie_id Infants Toddlers Schoolers
      save fam_char_pop.dta, replace
    restore

    merge m:1 pnr using e:/data/rawdata/707676/bef2019.dta, ///
              nogen keep(1 3) keepus(familie_id)
    merge m:1 familie_id using fam_char_pop.dta, nogen keep(1 3)

    * top code number of children
    replace Infants=3 if Infants>3
    replace Toddlers=3 if Toddlers>3
    replace Schoolers=3 if Schoolers>3

    * add socioeconomic status
    merge 1:m pnr using e:/data/rawdata/707676/ras2018.dta, ///
              nogen keep(1 3) keepus(soc* prim*)
    replace prim=99 if prim==0
    bysort pnr (primaer_status_kode): keep if _n==1
    ta prim
    ge InJob=inrange(soc_status_kode,110,138)
    ge Unemployed=soc_status_kode==200
    ge OLF=inrange(soc_status_kode,300,612)
    merge 1:1 pnr using e:/data/rawdata/707676/bef2019.dta, nogen keep(3) keepus(koen
  familie_id)
    // gender missing in pop data from Stat. Denmark
    merge m:1 familie_id using fam_char.dta, nogen keep(1 3)

    ge Male=koen==1
    ge Female=koen==2

    ge Age1829=aldergrp==1
    ge Age3039=aldergrp==2
    ge Age4049=aldergrp==3
    ge Age5059=aldergrp==4
    ge Age6069=aldergrp==5
    ge Age7079=aldergrp==6

    ge Single=inlist(famtype,1,2)
    ge Couple=inlist(famtype,3,4)

    ge NoChildren=HjembBoern==2
    ge Children=HjembBoern==1

    ge NorthernJutland=region==81
```

```
    ge CentralJutland=region==82
    ge SouthernJutland=region==83
    ge Capitol=region==84
    ge Zealand=region==85

    ge population=1
    keep  pnr population Male Female Age* ///
          Single Couple NoChildren Children Infants Toddlers Schoolers ///
          NorthernJutland CentralJutland SouthernJutland Capitol Zealand ///
          InJob Unemployed OLF
    save pop.dta, replace

    * append survey and population
    use panel_unbalanced_march_july.dta if time<3 & WHO5!=. & WSAS!=., clear
    ge population=0
    keep  pnr population Male Female Age* ///
          Single Couple NoChildren Children Infants Toddlers Schoolers ///
          NorthernJutland CentralJutland SouthernJutland Capitol Zealand ///
          InJob Unemployed OLF
    append using pop.dta
    save sample_pop_comparison.dta, replace

    * Table 1
    use sample_pop_comparison.dta
    local n=0
    foreach v in Male Female ///
               Age1829 Age3039 Age4049 Age5059 Age6069 Age7079 ///
               Single Couple ///
               NoChildren Children ///
               Infants Toddlers Schoolers ///
               NorthernJutland CentralJutland SouthernJutland Capitol Zealand ///
               InJob Unemployed OLF{
      preserve
          ttest `v', by(population)
          ge Variable="`v'"
          ge Survey=r(mu_1)
          ge Population=r(mu_2)
          ge sd_Survey=r(sd_1)
          ge sd_Population=r(sd_2)
          ge t=r(t)
          ge p=r(p)
          keep if _n==1
          keep Variable Survey sd_Survey Population sd_Population t p
          order Variable Survey sd_Survey Population sd_Population t p
          local n=`n'+1
          ge n=`n'
          if `n'>1 append using SurveyPopTest.dta
          sort n
          save SurveyPopTest.dta, replace
      restore
    }
    qui su population if population==0
    local Ns=r(N)
    qui su population if population==1
    local Np=r(N)

    use SurveyPopTest.dta, clear
    ge N_Survey=`Ns'
    ge N_Population=`Np'
    export excel using Table1.xlsx, firstrow(variables) replace


  ////////////////////////////////////////////////////////////////////////////////
  //  Table A2: Parameter estimates from OLS regressions of being at risk
  //            for depression/stress (WHO-5 < 50) and experiencing
  //            significant functional impairment (WSAS > 10)
  ////////////////////////////////////////////////////////////////////////////////

    * Define control variables
    glo X1 "Female Age1829 Age3039 Age5059 Age6069 Age7079"
    glo X2 "Single Children Infants Toddlers Schoolers InJob Unemp NorthernJutland
  CentralJutland SouthernJutland Zealand"

    * WHO-5 < 50
    use pnr int_dato time time1 time2 time3 WHO5 WHO5_50 WSAS WSAS_10 $X1 $X2 ///
```

```
        using panel_unbalanced_march_july.dta if WHO5!=. & WSAS!=., clear
    drop if time==3

    * Measure against time1
    replace time1=1

    qui reg WHO5_50 time1 time2 $X1, nocons vce(cl pnr)
    eststo WHO5_1
    qui reg WHO5_50 time1 time2 $X1 $X2, nocons vce(cl pnr)
    eststo WHO5_2

    * WSAS
    qui reg WSAS_10 time1 time2 $X1, nocons vce(cl pnr)
    eststo WSAS_1
    qui reg WSAS_10 time1 time2 $X1 $X2, nocons vce(cl pnr)
    eststo WSAS_2

    * print estimates table
    esttab WHO5_1 WHO5_2 WSAS_1 WSAS_2 ///
           using TableA2.rtf, se replace mtitles


  ////////////////////////////////////////////////////////////////////////////////
  //  Figure 2: Proportion of respondents at risk of depression or stress
  //            according to the WHO-5 and experiencing significant functional
  //            impairment according to the WSAS, by time of completing the
  //            survey relative to lockdown and by household structure
  ////////////////////////////////////////////////////////////////////////////////

    * WHO-5 < 50
    foreach v in Single Couple Children NoChildren{
      use pnr int_dato time* WHO5 WHO5_50 WSAS WSAS_10 `v' $X1 $X2 ///
          using panel_unbalanced_march_july.dta if WHO5!=. & WSAS!=., clear
      drop if time3==1

      keep if `v'==1

      qui reg WHO5_50 time1 time2 $X1, nocons vce(cl pnr)
      test time1=time2
      local F=r(F)
      local df=r(df)
      local p=r(p)
      margins, atmeans at(time1==1 time2==0) post
      ge b1=_b[_cons]
      ge ll1=b1-1.96*_se[_cons]
      ge ul1=b1+1.96*_se[_cons]
      qui reg WHO5_50 time1 time2 $X1, nocons vce(cl pnr)
      margins, atmeans at(time1==0 time2==1) post
      ge b2=_b[_cons]
      ge ll2=b2-1.96*_se[_cons]
      ge ul2=b2+1.96*_se[_cons]
      keep b1 ll1 ul1 b2 ll2 ul2
      keep if _n==1
      ge n=1
      reshape long b ll ul, i(n) j(time)
      drop n

      * export for plot
      export excel using Figure2_WHO5_`v'.xlsx, replace firstrow(variables)

      * stata plot
      two bar b time, fcolor(white) lcolor(gs1) barwidth(.45) || ///
          rcap ul ll time, lcolor(gs1) ///
          xlabel(1 "Before lockdown" 2 "During lockdown") ///
          xtitle("") ///
          ylabel(0(.1).4) ///
          ytitle("") ///
          title("`v'") ///
          legend(off) ///
          name(Figure2_WHO5_`v', replace)
    }

    * WSAS > 10
    foreach v in Single Couple Children NoChildren{
      use pnr int_dato time* WHO5 WHO5_50 WSAS WSAS_10 `v' $X1 $X2 ///
```

```
            using panel_unbalanced_march_july.dta if WHO5!=. & WSAS!=., clear
        drop if time3==1

        keep if `v'==1

        qui reg WSAS_10 time1 time2 $X1, nocons vce(cl pnr)
        test time1=time2
        local F=r(F)
        local df=r(df)
        local p=r(p)
        margins, atmeans at(time1==1 time2==0) post
        ge b1=_b[_cons]
        ge ll1=b1-1.96*_se[_cons]
        ge ul1=b1+1.96*_se[_cons]
        qui reg WSAS_10 time1 time2 $X1, nocons vce(cl pnr)
        margins, atmeans at(time1==0 time2==1) post
        ge b2=_b[_cons]
        ge ll2=b2-1.96*_se[_cons]
        ge ul2=b2+1.96*_se[_cons]
        keep b1 ll1 ul1 b2 ll2 ul2
        keep if _n==1
        ge n=1
        reshape long b ll ul, i(n) j(time)
        drop n

        * export for plot
        export excel using Figure2_WSAS_`v'.xlsx, replace firstrow(variables)

        * stata plot
        two bar b time, fcolor(white) lcolor(gs1) barwidth(.45) || ///
            rcap ul ll time, lcolor(gs1) ///
            xlabel(1 "Before lockdown" 2 "During lockdown") ///
            xtitle("") ///
            ylabel(0(.1).4) ///
            ytitle("") ///
            title("`v'") ///
            legend(off) ///
            name(Figure2_WSAS_`v', replace)
    }

    * Set up Figure A1
    graph combine Figure2_WHO5_Single Figure2_WHO5_Couple ///
                  Figure2_WHO5_NoChildren Figure2_WHO5_Children ///
                  Figure2_WSAS_Single Figure2_WSAS_Couple ///
                  Figure2_WSAS_NoChildren Figure2_WSAS_Children ///
                  , iscale(1) altshrink title("Early vs. Late March 2020")
    graph export Figure2.png, replace


  ////////////////////////////////////////////////////////////////////////////////
  //  Table A3: Parameter estimates from OLS regressions of being at risk of
  //            depression/stress (WHO-5 < 50), by household structure
  //
  //  Table A4: Parameter estimates from OLS regressions of being at risk of
  //            significant functional impairment (WSAS > 10), by household
  //            structure
  //
  //  + Chow tests of subgroup differences
  ////////////////////////////////////////////////////////////////////////////////

    * WHO-5 < 50
    use pnr int_dato time* WHO5 WHO5_50 WSAS WSAS_10 Single Couple NoChildren ///
    Children $X1 using panel_unbalanced_march_july.dta if WHO5!=. & WSAS!=., clear

    drop if time==3

    * Measure against time1
    replace time1=1

    * WHO-5 < 50
    qui reg WHO5_50 time1 time2 $X1, nocons vce(cl pnr), if Single==1
    eststo WHO5_single
    qui reg WHO5_50 time1 time2 $X1, nocons vce(cl pnr), if Couple==1
    eststo WHO5_couple
    qui reg WHO5_50 time1 time2 $X1, nocons vce(cl pnr), if NoChildren==1
```

```
    eststo WHO5_nokids
    qui reg WHO5_50 time1 time2 $X1, nocons vce(cl pnr), if Children==1
    eststo WHO5_kids

    * WSAS > 10
    qui reg WSAS_10 time1 time2 $X1, nocons vce(cl pnr), if Single==1
    eststo WSAS_single
    qui reg WSAS_10 time1 time2 $X1, nocons vce(cl pnr), if Couple==1
    eststo WSAS_couple
    qui reg WSAS_10 time1 time2 $X1, nocons vce(cl pnr), if NoChildren==1
    eststo WSAS_nokids
    qui reg WSAS_10 time1 time2 $X1, nocons vce(cl pnr), if Children==1
    eststo WSAS_kids

    * print estimates Table A3 and Table A5
    esttab WHO5_single WHO5_couple WHO5_nokids WHO5_kids ///
           using TableA3.rtf, se replace mtitles
    esttab WSAS_single WSAS_couple WSAS_nokids WSAS_kids ///
           using TableA4.rtf, se replace mtitles

    * Chow test subgroups using SUEST
    use pnr int_dato time* WHO5 WHO5_50 WSAS WSAS_10 Single Couple NoChildren ///
    Children $X1 using panel_unbalanced_march_july.dta if WHO5!=. & WSAS!=., clear

    drop if time3==1

    qui reg WHO5_50 time2 $X1 if Single==1
    est sto Sing
    qui reg WHO5_50 time2 $X1 if Couple==1
    est sto NoSing

    suest Sing NoSing
    test  [Sing_mean]time2 - [NoSing_mean]time2 = 0

    qui reg WSAS_10 time2 $X1 if Single==1
    est sto Sing
    qui reg WSAS_10 time2 $X1 if Couple==1
    est sto NoSing

    suest Sing NoSing
    test  [Sing_mean]time2 - [NoSing_mean]time2 = 0

    qui reg WHO5_50 time2 $X1 if Children==1
    est sto Kids
    qui reg WHO5_50 time2 $X1 if NoChildren==1
    est sto NoKids

    suest Kids NoKids, vce(robust)
    test  [Kids_mean]time2 - [NoKids_mean]time2 = 0

    qui reg WSAS_10 time2 $X1 if Children==1
    est sto Kids
    qui reg WSAS_10 time2 $X1 if NoChildren==1
    est sto NoKids

    suest Kids NoKids, vce(robust)
    test  [Kids_mean]time2 - [NoKids_mean]time2 = 0


  ////////////////////////////////////////////////////////////////////////////////
  //  Table A5: Parameter estimates from OLS regressions of being at risk of
  //            depression/stress (WHO-5 < 50) and experiencing significant
  //            functional impairment (WSAS > 10), repeat responses in early
  //            March and July
  ////////////////////////////////////////////////////////////////////////////////

    * WHO-5 < 50
    use pnr int_dato time* WHO5 WHO5_50 WSAS WSAS_10 $X1 $X2 ///
    using panel_balanced_march_july.dta if WHO5!=. & WSAS!=., clear

    bysort pnr: egen q=max(time2)
    drop if q==1

    * Measure against time1
    replace time1=1
```

```
    qui reg WHO5_50 time1 time3 $X1, nocons vce(cl pnr)
    eststo WHO5_1
    qui reg WHO5_50 time1 time3 $X1 $X2, nocons vce(cl pnr)
    eststo WHO5_2

    * WSAS > 10
    qui reg WSAS_10 time1 time3 $X1, nocons vce(cl pnr)
    eststo WSAS_1
    qui reg WSAS_10 time1 time3 $X1 $X2, nocons vce(cl pnr)
    eststo WSAS_2

    * print estimates table
    esttab WHO5_1 WHO5_2 WSAS_1 WSAS_2 using TableA5.rtf, se replace mtitles


  ////////////////////////////////////////////////////////////////////////////////
  //  Figure 3: Proportion of respondents at risk of depression or stress
  //            according to the WHO-5, repeat responses in early March and July,
  //            by household structure
  ////////////////////////////////////////////////////////////////////////////////

    * WHO5 < 50
    foreach v in Single Couple NoChildren Children{
      use pnr int_dato time* WHO5 WHO5_50 WSAS `v' $X1 $X2 ///
          using panel_balanced_march_july.dta if WHO5!=. & WSAS!=., clear
      bysort pnr: egen q=max(time2)
      drop if q==1

      keep if `v'==1

      qui reg WHO5_50 time1 time3 $X1, nocons vce(cl pnr)
      test time1=time3
      local F=r(F)
      local df=r(df)
      local p=r(p)
      margins, atmeans at(time1==1 time3==0) post
      ge b1=_b[_cons]
      ge ll1=b1-1.96*_se[_cons]
      ge ul1=b1+1.96*_se[_cons]
      qui reg WHO5_50 time1 time3 $X1, nocons vce(cl pnr)
      margins, atmeans at(time1==0 time3==1) post
      ge b2=_b[_cons]
      ge ll2=b2-1.96*_se[_cons]
      ge ul2=b2+1.96*_se[_cons]
      keep b1 ll1 ul1 b2 ll2 ul2
      keep if _n==1
      ge n=1
      reshape long b ll ul, i(n) j(time)
      drop n

      * export for plotting in R
      export excel using Figure3_WHO5_`v'.xlsx, replace firstrow(variables)

      * stata plot
      two bar b time, fcolor(white) lcolor(gs1) barwidth(.45) || ///
        rcap ul ll time, lcolor(gs1) ///
        xlabel(1 "Early March" 2 "July") ///
        xtitle("") ///
        ylabel(0(.1).4) ///
        ytitle("") ///
        title("`v'") ///
        legend(off) ///
        name(Figure3_WHO5_`v', replace)
  }

    * Figure 3: Upper panel
    graph combine Figure3_WHO5_Single Figure3_WHO5_Couple ///
                  Figure3_WHO5_NoChildren Figure3_WHO5_Children ///
                  , iscale(1) altshrink title("At risk of stress or depression, WHO5<50")
    graph export Figure3_WHO5.png, replace


    * WSAS > 10
    foreach v in Single Couple NoChildren Children{
```

```
      use pnr int_dato time* WHO5 WSAS WSAS_10 `v' $X1 $X2 ///
          using panel_balanced_march_july.dta if WHO5!=. & WSAS!=., clear

      bysort pnr: egen q=max(time2)
      drop if q==1

      keep if `v'==1

      qui reg WSAS_10 time1 time3 $X1, nocons vce(cl pnr)
      test time1=time3
      local F=r(F)
      local df=r(df)
      local p=r(p)
      margins, atmeans at(time1==1 time3==0) post
      ge b1=_b[_cons]
      ge ll1=b1-1.96*_se[_cons]
      ge ul1=b1+1.96*_se[_cons]
      qui reg WSAS_10 time1 time3 $X1, nocons vce(cl pnr)
      margins, atmeans at(time1==0 time3==1) post
      ge b2=_b[_cons]
      ge ll2=b2-1.96*_se[_cons]
      ge ul2=b2+1.96*_se[_cons]
      keep b1 ll1 ul1 b2 ll2 ul2
      keep if _n==1
      ge n=1
      reshape long b ll ul, i(n) j(time)
      drop n

      * export for plotting in R
      export excel using Figure3_WSAS_`v'.xlsx, replace firstrow(variables)

      * stata plot
      two bar b time, fcolor(white) lcolor(gs1) barwidth(.45) || ///
        rcap ul ll time, lcolor(gs1) ///
        xlabel(1 "Early March" 2 "July") ///
        xtitle("") ///
        ylabel(0(.1).4) ///
        ytitle("") ///
        title("`v'") ///
        legend(off) ///
        name(Figure3_WSAS_`v', replace)
    }

    * Lower panel of Figure 3
    graph combine Figure3_WSAS_Single Figure3_WSAS_Couple ///
                  Figure3_WSAS_NoChildren Figure3_WSAS_Children ///
                  , iscale(1) altshrink title("Significantly functionally impaired, WSAS>10")
    graph export Figure3_WSAS.png, replace


  ////////////////////////////////////////////////////////////////////////////////
  //  Table A6: Parameter estimates from OLS regressions of being at risk of
  //            depression/stress (WHO-5 < 50), by household structure. Repeat
  //            responses in early March and July
  //
  //  Table A7: Parameter estimates from OLS regressions of experiencing
  //            significant functional impairment (WSAS > 10),
  //            by household structure. Repeat responses in early March and July
  ////////////////////////////////////////////////////////////////////////////////

    use pnr int_dato time* WHO5 WHO5_50 WSAS WSAS_10 ///
        Single Couple NoChildren Children $X1 ///
        using panel_unbalanced_march_july.dta if WHO5!=. & WSAS!=., clear

    bysort pnr: egen q=max(time2)
    drop if q==1

    * Measure against time1
    replace time1=1

    * WHO-5 < 50
    qui reg WHO5_50 time1 time3 $X1, nocons vce(cl pnr), if Single==1
    eststo WHO5_single
    qui reg WHO5_50 time1 time3 $X1, nocons vce(cl pnr), if Couple==1
    eststo WHO5_couple
```

```
    qui reg WHO5_50 time1 time3 $X1, nocons vce(cl pnr), if Children==1
    eststo WHO5_kids
    qui reg WHO5_50 time1 time3 $X1, nocons vce(cl pnr), if NoChildren==1
    eststo WHO5_nokids

    * Print Table A6
    esttab WHO5_single WHO5_couple WHO5_nokids WHO5_kids ///
           using TableA6.rtf, se replace mtitles

    * WSAS > 10
    qui reg WSAS_10 time1 time3 $X1, nocons vce(cl pnr), if Single==1
    eststo WSAS_single
    qui reg WSAS_10 time1 time3 $X1, nocons vce(cl pnr), if Couple==1
    eststo WSAS_couple
    reg WSAS_10 time1 time3 $X1, nocons vce(cl pnr), if Children==1
    eststo WSAS_kids
    qui reg WSAS_10 time1 time3 $X1, nocons vce(cl pnr), if NoChildren==1
    eststo WSAS_nokids

    * Print Table A7
    esttab WSAS_single WSAS_couple WSAS_nokids WSAS_kids ///
           using TableA7.rtf, se replace mtitles


////////////////////////////////////////////////////////////////////////////////
//  Figure A1: Mean score on subdomains of the WSAS, by time of completing
//              the survey. Respondents with children living at home
////////////////////////////////////////////////////////////////////////////////

    use pnr int_dato time* WHO5 WSAS C1 C2 C3 C4 C5 Children $X1 ///
        using panel_balanced_march_july.dta if WHO5!=. & WSAS!=., clear

    drop if time3==1
    keep if Children==1

    rename (C1 C2 C3 C4 C5) (Job Home Social Leisure Family)

    foreach y in Job Home Social Leisure Family{
      preserve
        qui reg `y' time1 time2 $X1, nocons vce(cl pnr)
        margins, atmeans at(time1==1 time2==0) post
        ge b1=_b[_cons]
        ge ll1=b1-1.96*_se[_cons]
        ge ul1=b1+1.96*_se[_cons]
        qui reg `y' time1 time2 $X1, nocons vce(cl pnr)
        margins, atmeans at(time1==0 time2==1) post
        ge b2=_b[_cons]
        ge ll2=b2-1.96*_se[_cons]
        ge ul2=b2+1.96*_se[_cons]

        keep b1 ul1 ll1 b2 ul2 ll2
        keep if _n==1
        ge n=1
        reshape long b ll ul, i(n) j(time)
        drop n

        * export for plotting in R
        export excel using FigureA1_`y'.xlsx, replace firstrow(variables)

        * stata plot
        two bar b time, fcolor(white) lcolor(gs1) barwidth(.45) || ///
        rcap ul ll time, lcolor(gs1) ///
        xlabel(1 "Before lockdown" 2 "During lockdown March") ///
        xtitle("") ///
        ylabel(0(.25)1.5) ///
        ytitle("Share") ///
        legend(off) ///
        title("`y'") ///
        name(FigA1_`y', replace)
      restore
    }

    * combine graphs to FigureA1
    graph combine FigA1_Job FigA1_Home FigA1_Social FigA1_Leisure FigA1_Family ///
                  , iscale(1) cols(2) ///
```

```
                    altshrink title("Mean score on subdomains of the WSAS") ///
                    subtitle("Scales are 0-8")
    graph export FigureA1.png, replace


  ///////////////////////////////////////////////////////////////////////////////
  //  Figure A2: Proportion of respondents at risk of depression or stress
  //             according to the WHO-5 and proportion of respondents experiencing
  //             significant functional impairment according to the WSAS, by time
  //             of completing the survey and including respndents in late March
  ///////////////////////////////////////////////////////////////////////////////


    * WHO5 < 50
    use pnr int_dato time* WHO5 WHO5_50 WSAS $X1 ///
        using panel_balanced_march_july.dta if WHO5!=. & WSAS!=., clear

    qui reg WHO5_50 time1 time2 time3 $X1, nocons vce(cl pnr)
    margins, atmeans at(time1==1 time2==0 time3==0) post
    ge b1=_b[_cons]
    ge ll1=b1-1.96*_se[_cons]
    ge ul1=b1+1.96*_se[_cons]
    qui reg WHO5_50 time1 time2 time3 $X1, nocons vce(cl pnr)
    margins, atmeans at(time1==0 time2==1 time3==0) post
    ge b2=_b[_cons]
    ge ll2=b2-1.96*_se[_cons]
    ge ul2=b2+1.96*_se[_cons]
    qui reg WHO5_50 time1 time2 time3 $X1, nocons vce(cl pnr)
    margins, atmeans at(time1==0 time2==0 time3==1) post
    ge b3=_b[_cons]
    ge ll3=b3-1.96*_se[_cons]
    ge ul3=b3+1.96*_se[_cons]

    keep b1 ll1 ul1 b2 ll2 ul2 b3 ll3 ul3
    keep if _n==1
    ge n=1
    reshape long b ll ul, i(n) j(time)
    drop n

    * export for plotting in R
    export excel using FigureA2_WHO5.xlsx, replace firstrow(variables)

    * stata plot
    replace time=1.5 if time==2
    replace time=2.5 if time==3
    two bar b time, fcolor(white) lcolor(gs1) barwidth(.45) || ///
        rcap ul ll time, lcolor(gs1) ///
        xlabel(1 "Early March" 1.5 "Late March" 2.5  "July") ///
        xtitle("") ///
        ylabel(0(.1).4) ///
        ytitle("") ///
        legend(off) ///
        title("At risk of depression or stress (WHO5<50)") ///
        name(FigA2_WHO5, replace)


    * WSAS > 10
    use pnr int_dato time* WHO5 WSAS WSAS_10 $X1 ///
        using panel_balanced_march_july.dta if WHO5!=. & WSAS!=., clear

    qui reg WSAS_10 time1 time2 time3 $X1, nocons vce(cl pnr)
    margins, atmeans at(time1==1 time2==0 time3==0) post
    ge b1=_b[_cons]
    ge ll1=b1-1.96*_se[_cons]
    ge ul1=b1+1.96*_se[_cons]
    qui reg WSAS_10 time1 time2 time3 $X1, nocons vce(cl pnr)
    margins, atmeans at(time1==0 time2==1 time3==0) post
    ge b2=_b[_cons]
    ge ll2=b2-1.96*_se[_cons]
    ge ul2=b2+1.96*_se[_cons]
    qui reg WSAS_10 time1 time2 time3 $X1, nocons vce(cl pnr)
    margins, atmeans at(time1==0 time2==0 time3==1) post
    ge b3=_b[_cons]
    ge ll3=b3-1.96*_se[_cons]
    ge ul3=b3+1.96*_se[_cons]
```

```

    keep b1 ll1 ul1 b2 ll2 ul2 b3 ll3 ul3
    keep if _n==1
    ge n=1
    reshape long b ll ul, i(n) j(time)
    drop n

    * export for plotting in R
    export excel using FigureA2_WSAS.xlsx, replace firstrow(variables)

    * stata plot
     replace time=1.5 if time==2
    replace time=2.5 if time==3
    two bar b time, fcolor(white) lcolor(gs1) barwidth(.45) || ///
        rcap ul ll time, lcolor(gs1) ///
        xlabel(1 "Early March" 1.5 "Late March" 2.5  "July") ///
        xtitle("") ///
        ylabel(0(.1).4) ///
        ytitle("") ///
        legend(off) ///
        title("Significant functional impairment (WSAS>10)") ///
        name(FigA2_WSAS, replace)

    * combine graphs to get Figure A4
    graph combine FigA2_WHO5 FigA2_WSAS, ///
          iscale(1) altshrink ///
          title("WHO-5 < 50 and WSAS > 10, Early March, late March and July")
    graph export FigureA2.png, replace


  ///////////////////////////////////////////////////////////////////////////////////
  //  Figure A3: Proportion of respondents at risk of depression or stress
  //            according to the WHO-5, by time of completing the survey and by
  //            household structure; including respondents in late March
  ///////////////////////////////////////////////////////////////////////////////////

    * WHO5 < 50
    foreach v in Single Couple NoChildren Children{
      use pnr int_dato time* WHO5 WHO5_50 WSAS `v' $X1 ///
          using panel_balanced_march_july.dta if WHO5!=. & WSAS!=., clear

    keep if `v'==1

    qui reg WHO5_50 time1 time2 time3 $X1, nocons vce(cl pnr)
    margins, atmeans at(time1==1 time2==0 time3==0) post
    ge b1=_b[_cons]
    ge ll1=b1-1.96*_se[_cons]
    ge ul1=b1+1.96*_se[_cons]
    qui reg WHO5_50 time1 time2 time3 $X1, nocons vce(cl pnr)
    margins, atmeans at(time1==0 time2==1 time3==0) post
    ge b2=_b[_cons]
    ge ll2=b2-1.96*_se[_cons]
    ge ul2=b2+1.96*_se[_cons]
    qui reg WHO5_50 time1 time2 time3 $X1, nocons vce(cl pnr)
    margins, atmeans at(time1==0 time2==0 time3==1) post
    ge b3=_b[_cons]
    ge ll3=b3-1.96*_se[_cons]
    ge ul3=b3+1.96*_se[_cons]
    keep b1 ll1 ul1 b2 ll2 ul2 b3 ll3 ul3
    keep if _n==1
    ge n=1
    reshape long b ll ul, i(n) j(time)
    drop n

    * export for plotting in R
    export excel using FigureA3_WHO5_`v'.xlsx, replace firstrow(variables)

    * stata plot
    replace time=1.5 if time==2
    replace time=2.5 if time==3
    two bar b time, fcolor(white) lcolor(gs1) barwidth(.45) || ///
        rcap ul ll time, lcolor(gs1) ///
        xlabel(1 "Early March" 1.5 "Late March" 2.5  "July") ///
        xtitle("") ///
        ylabel(0(.1).4) ///
```

```
		ytitle("") ///
		title("`v'") ///
		legend(off) ///
		name(FigureA3_WHO5_`v', replace)
	}

	* Print Figure A3
	graph combine FigureA3_WHO5_Single FigureA3_WHO5_Couple ///
				FigureA3_WHO5_NoChildren FigureA3_WHO5_Children ///
				, iscale(1) altshrink ///
				title("At risk of stress or depression, WHO-5 < 50")
	graph export FigureA3.png, replace


////////////////////////////////////////////////////////////////////////////////
//  Figure A4: Proportion of respondents experiencing significant functional
//             impairment according to the WSAS, by time of completing the
//             survey and by household structure; including respondents in
//             late March
////////////////////////////////////////////////////////////////////////////////

	* WSAS > 10
	foreach v in Single Couple NoChildren Children{
	  use pnr int_dato time* WHO5 WSAS WSAS_10 `v' $X1 ///
	      using panel_balanced_march_july.dta if WHO5!=. & WSAS!=., clear

	keep if `v'==1

	qui reg WSAS_10 time1 time2 time3 $X1, nocons vce(cl pnr)
	margins, atmeans at(time1==1 time2==0 time3==0) post
	ge b1=_b[_cons]
	ge ll1=b1-1.96*_se[_cons]
	ge ul1=b1+1.96*_se[_cons]
	qui reg WSAS_10 time1 time2 time3 $X1, nocons vce(cl pnr)
	margins, atmeans at(time1==0 time2==1 time3==0) post
	ge b2=_b[_cons]
	ge ll2=b2-1.96*_se[_cons]
	ge ul2=b2+1.96*_se[_cons]
	qui reg WSAS_10 time1 time2 time3 $X1, nocons vce(cl pnr)
	margins, atmeans at(time1==0 time2==0 time3==1) post
	ge b3=_b[_cons]
	ge ll3=b3-1.96*_se[_cons]
	ge ul3=b3+1.96*_se[_cons]
	keep b1 ll1 ul1 b2 ll2 ul2 b3 ll3 ul3
	keep if _n==1
	ge n=1
	reshape long b ll ul, i(n) j(time)
	drop n

	* export for plotting in R
	export excel using FigureA4_WSAS_`v'.xlsx, replace firstrow(variables)

	* stata plot
	replace time=1.5 if time==2
	replace time=2.5 if time==3
	two bar b time, fcolor(white) lcolor(gs1) barwidth(.45) || ///
		rcap ul ll time, lcolor(gs1) ///
		xlabel(1 "Early March" 1.5 "Late March" 2.5  "July") ///
		xtitle("") ///
		ylabel(0(.1).4) ///
		ytitle("") ///
		title("`v'") ///
		legend(off) ///
		name(FigureA4_WSAS_`v', replace)
	}

	* Print Figure A4
	graph combine FigureA4_WSAS_Single FigureA4_WSAS_Couple ///
				FigureA4_WSAS_NoChildren FigureA4_WSAS_Children ///
				, iscale(1) altshrink ///
				title("Significantly functionally impaired, WSAS > 10")
	graph export FigureA4.png, replace


////////////////////////////////////////////////////////////////////////////////
```

```
//  Table A8: Summary of robustness check of results' sensitivity to cutoffs
//            on the WHO-5 and WSAS scales
////////////////////////////////////////////////////////////////////////////////

  use pnr int_dato time* WHO5 WHO5_13 WSAS $X1 $X2 ///
      using panel_unbalanced_march_july.dta if WHO5!=. & WSAS!=., clear

  drop if time==3

  * Measure against time1
  replace time1=1

  * WHO-5
  foreach v in 13 40 45 50 55 60{
    if `v'!=13 ge WHO5_`v'=WHO5<`v'
    qui reg WHO5_`v' time1 time2 $X1, nocons vce(cl pnr)
    eststo WHO5_`v'
  }

  * WSAS
  foreach v in 5 8 10 12 15 20{
    ge WSAS_`v'=WSAS>`v'
    qui reg WSAS_`v' time1 time2 $X1, nocons vce(cl pnr)
    eststo WSAS_`v'
  }

  * print estimates table
  esttab WHO5_13 WHO5_40 WHO5_45 WHO5_50 WHO5_55 WHO5_60 ///
       using TableA8.rtf, se replace mtitles ///
       keep(time1 time2) nonotes
  esttab WSAS_5 WSAS_8 WSAS_10 WSAS_12 WSAS_15 WSAS_20 ///
       using TableA8.rtf, se append mtitles ///
       keep(time1 time2) ///
       addnotes("All models controlled for gender and age." ///
                "WHO5<50 signals increased risk of depression and/or stress." ///
                "WHO5 raw score <13 signals very poor wellbeing." ///
                "WSAS>10 signals significant functional impairment." ///
                "WSAS>20 signals moderately severe or worse psychopathology.")


////////////////////////////////////////////////////////////////////////////////
//  Figure A5: Main results but using population weights from Statistics
//             Denmark instead of statistical controlling
////////////////////////////////////////////////////////////////////////////////

  * WHO5 < 50
  use pnr time* WHO5 WHO5_50 WSAS pervgt ///
      using panel_unbalanced_march_july.dta if WHO5!=. & WSAS!=., clear

  drop if time3==1

  qui reg WHO5_50 time1 time2 [aw=pervgt], nocons vce(cl pnr)
  test time1=time2
  local F=r(F)
  local df=r(df)
  local p=r(p)
  margins, atmeans at(time1==1 time2==0) post
  ge b1=_b[_cons]
  ge ll1=b1-1.96*_se[_cons]
  ge ul1=b1+1.96*_se[_cons]
  qui reg WHO5_50 time1 time2 [aw=pervgt], nocons vce(cl pnr)
  margins, atmeans at(time1==0 time2==1) post
  ge b2=_b[_cons]
  ge ll2=b2-1.96*_se[_cons]
  ge ul2=b2+1.96*_se[_cons]
  keep b1 ll1 ul1 b2 ll2 ul2
  keep if _n==1
  ge n=1
  reshape long b ll ul, i(n) j(time)
  drop n

  * export for plotting in R
  export excel using FigureA5_WHO5.xlsx, replace firstrow(variables)

  * stata plot
```

```
    two bar b time, fcolor(white) lcolor(gs1) barwidth(.45) || ///
        rcap ul ll time, lcolor(gs1) ///
        xlabel(1 "Before lockdown" 2 "During lockdown March") ///
        xtitle("") ///
        ylabel(0(.1).4) ///
        ytitle("Share") ///
        legend(off) ///
        title("At risk of depression or stress (WHO5<50)") ///
        name(FigA5_WHO5, replace)

    * WSAS > 10
    use pnr time* WHO5 WSAS WSAS_10 pervgt ///
        using panel_unbalanced_march_july.dta if WHO5!=. & WSAS!=., clear

    drop if time3==1

    qui reg WSAS_10 time1 time2 [aw=pervgt], nocons vce(cl pnr)
    test time1=time2
    local F=r(F)
    local df=r(df)
    local p=r(p)
    margins, atmeans at(time1==1 time2==0) post
    ge b1=_b[_cons]
    ge ll1=b1-1.96*_se[_cons]
    ge ul1=b1+1.96*_se[_cons]
    qui reg WSAS_10 time1 time2 [aw=pervgt], nocons vce(cl pnr)
    margins, atmeans at(time1==0 time2==1) post
    ge b2=_b[_cons]
    ge ll2=b2-1.96*_se[_cons]
    ge ul2=b2+1.96*_se[_cons]
    keep b1 ll1 ul1 b2 ll2 ul2
    keep if _n==1
    ge n=1
    reshape long b ll ul, i(n) j(time)
    drop n

    * export for plotting in R
    export excel using FigureA5_WSAS.xlsx, replace firstrow(variables)

    * stata plot
    two bar b time, fcolor(white) lcolor(gs1) barwidth(.45) || ///
        rcap ul ll time, lcolor(gs1) ///
        xlabel(1 "Before lockdown" 2 "During lockdown March") ///
        xtitle("") ///
        ylabel(0(.1).4) ///
        ytitle("Share") ///
        legend(off) ///
        title("Significant functional impairment (WSAS>10)") ///
        name(FigA5_WSAS, replace)

    * combine and export Figure A5
    graph combine FigA5_WHO5 FigA5_WSAS ///
                , iscale(1) altshrink ///
                title("Main results weighted using" ///
                      "population weights from Statistics Denmark")
    graph export FigureA5.png, replace


  ////////////////////////////////////////////////////////////////////////////////
  //  Figure A6: Proportion of respondents at risk of depression or stress
  //             according to the WHO-5, comparing main results and main results
  //             by household structure to results down weighted respondents
  //             March 12 -- March 25
  ////////////////////////////////////////////////////////////////////////////////

    use pnr int_dato time* WHO5 WHO5_50 WSAS $X1 ///
        using panel_unbalanced_march_july.dta if WHO5!=. & WSAS!=., clear

    drop if time3==1

    * define WHO-5 weights from response date relative to March 11
    ge datedif=int_dato-mdy(3,11,2020)
    replace datedif=datedif+1 if datedif>=0
    // +1 so those responded on March 11 counts as just after
    ta datedif
```

```
	ge w_WHO5=15
	replace w_WHO5=datedif if inrange(datedif,0,14)
	ta w_WHO5
	replace w_WHO5=w_WHO5/15
	ta w_WHO5

	qui reg WHO5_50 time1 time2 $X1 [aw=w_WHO], nocons vce(cl pnr)
	test time1=time2
	local F=r(F)
	local df=r(df)
	local p=r(p)
	margins, atmeans at(time1==1 time2==0) post
	ge b1=_b[_cons]
	ge ll1=b1-1.96*_se[_cons]
	ge ul1=b1+1.96*_se[_cons]
	qui reg WHO5_50 time1 time2 $X1 [aw=w_WHO], nocons vce(cl pnr)
	margins, atmeans at(time1==0 time2==1) post
	ge b2=_b[_cons]
	ge ll2=b2-1.96*_se[_cons]
	ge ul2=b2+1.96*_se[_cons]
	keep b1 ll1 ul1 b2 ll2 ul2
	keep if _n==1
	ge n=1
	reshape long b ll ul, i(n) j(time)
	drop n

	* export for plotting in R
	export excel using FigureA6_weighted.xlsx, replace firstrow(variables)

	* stata plot
	two bar b time, fcolor(white) lcolor(gs1) barwidth(.45) || ///
		rcap ul ll time, lcolor(gs1) ///
		xlabel(1 "Before lockdown" 2 "During lockdown March") ///
		xtitle("") ///
		ylabel(0(.1).4) ///
		ytitle("") ///
		legend(off) ///
		title("March 12 - March 25 downweighted") ///
		name(FigA6_w_all, replace)

	* rerun main results
	use pnr time* WHO5 WHO5_50 WSAS $X1 ///
		using panel_unbalanced_march_july.dta if WHO5!=. & WSAS!=., clear

	drop if time3==1

	qui reg WHO5_50 time1 time2 $X1, nocons vce(cl pnr)
	test time1=time2
	local F=r(F)
	local df=r(df)
	local p=r(p)
	margins, atmeans at(time1==1 time2==0) post
	ge b1=_b[_cons]
	ge ll1=b1-1.96*_se[_cons]
	ge ul1=b1+1.96*_se[_cons]
	qui reg WHO5_50 time1 time2 $X1, nocons vce(cl pnr)
	margins, atmeans at(time1==0 time2==1) post
	ge b2=_b[_cons]
	ge ll2=b2-1.96*_se[_cons]
	ge ul2=b2+1.96*_se[_cons]
	keep b1 ll1 ul1 b2 ll2 ul2
	keep if _n==1
	ge n=1
	reshape long b ll ul, i(n) j(time)
	drop n

	* export for plotting in R
	export excel using FigureA6_main.xlsx, replace firstrow(variables)

	* stata plot
	two bar b time, fcolor(white) lcolor(gs1) barwidth(.45) || ///
		rcap ul ll time, lcolor(gs1) ///
		xlabel(1 "Before lockdown" 2 "During lockdown March") ///
		xtitle("") ///
		ylabel(0(.1).4) ///
```

```
        ytitle("") ///
        legend(off) ///
        title("All: Main results") ///
        name(FigA6_main_all, replace)

    foreach v in Single Couple NoChildren Children{
      use pnr int_dato time* WHO5 WHO5_50 WSAS `v' $X1 ///
          using panel_unbalanced_march_july.dta if WHO5!=. & WSAS!=., clear

      drop if time3==1
      keep if `v'==1

      * define WHO-5 weights from response date relative to March 11
      ge datedif=int_dato-mdy(3,11,2020)
      replace datedif=datedif+1 if datedif>=0
      // +1 so those responded on March 11 counts as just after
      ta datedif
      ge w_WHO5=15
      replace w_WHO5=datedif if inrange(datedif,0,14)
      ta w_WHO5
      replace w_WHO5=w_WHO5/15
      ta w_WHO5

      qui reg WHO5_50 time1 time2 $X1 [aw=w_WHO], nocons vce(cl pnr)
      test time1=time2
      local F=r(F)
      local df=r(df)
      local p=r(p)
      margins, atmeans at(time1==1 time2==0) post
      ge b1=_b[_cons]
      ge ll1=b1-1.96*_se[_cons]
      ge ul1=b1+1.96*_se[_cons]
      qui reg WHO5_50 time1 time2 $X1 [aw=w_WHO], nocons vce(cl pnr)
      margins, atmeans at(time1==0 time2==1) post
      ge b2=_b[_cons]
      ge ll2=b2-1.96*_se[_cons]
      ge ul2=b2+1.96*_se[_cons]
      keep b1 ll1 ul1 b2 ll2 ul2
      keep if _n==1
      ge n=1
      reshape long b ll ul, i(n) j(time)
      drop n

      * export for plotting in R
      export excel using FigureA6_weighted_`v'.xlsx, replace firstrow(variables)

      * stata plot
      two bar b time, fcolor(white) lcolor(gs1) barwidth(.45) || ///
        rcap ul ll time, lcolor(gs1) ///
        xlabel(1 "Before lockdown" 2 "During lockdown March") ///
        xtitle("") ///
        ylabel(0(.1).4) ///
        ytitle("") ///
        legend(off) ///
        title("March 12 - March 25 downweighted") ///
        name(FigA6_w_`v', replace)

      * rerun main results
      use pnr time* WHO5 WHO5_50 WSAS `v' $X1 ///
        using panel_unbalanced_march_july.dta if WHO5!=. & WSAS!=., clear

      drop if time3==1
      keep if `v'==1

      qui reg WHO5_50 time1 time2 $X1, nocons vce(cl pnr)
      test time1=time2
      local F=r(F)
      local df=r(df)
      local p=r(p)
      margins, atmeans at(time1==1 time2==0) post
      ge b1=_b[_cons]
      ge ll1=b1-1.96*_se[_cons]
      ge ul1=b1+1.96*_se[_cons]
      qui reg WHO5_50 time1 time2 $X1, nocons vce(cl pnr)
      margins, atmeans at(time1==0 time2==1) post
```

```
      ge b2=_b[_cons]
      ge ll2=b2-1.96*_se[_cons]
      ge ul2=b2+1.96*_se[_cons]
      keep b1 ll1 ul1 b2 ll2 ul2
      keep if _n==1
      ge n=1
      reshape long b ll ul, i(n) j(time)
      drop n

      * export for plotting in R
      export excel using FigureA6_main_`v'.xlsx, replace firstrow(variables)

      * stata plot
      two bar b time, fcolor(white) lcolor(gs1) barwidth(.45) || ///
        rcap ul ll time, lcolor(gs1) ///
        xlabel(1 "Before lockdown" 2 "During lockdown March") ///
        xtitle("") ///
        ylabel(0(.1).4) ///
        ytitle("") ///
        legend(off) ///
        title("`v': Main results") ///
        name(FigA6_main_`v', replace)
    }

    * combine to produce Figure A6
    graph combine FigA6_main_all FigA6_w_all ///
                  FigA6_main_Single FigA6_w_Single ///
                  FigA6_main_Couple FigA6_w_Couple ///
                  FigA6_main_NoChildren FigA6_w_NoChildren ///
                  FigA6_main_Children FigA6_w_Children ///
                , iscale(1) cols(2) ///
                altshrink title("At risk of depression or stress (WHO-5 < 50)")
    graph export FigureA6.png, replace


  ////////////////////////////////////////////////////////////////////////////////
  //  Figure A7: Proportion of respondents experiencing significant functional
  //             impairment according to the WSAS, comparing main results and main
  //             results by household structure to results down weighting
  //             respondents March 12 -- March 25
  ////////////////////////////////////////////////////////////////////////////////

    use pnr int_dato time* WHO5 WSAS WSAS_10 $X1 ///
        using panel_unbalanced_march_july.dta if WHO5!=. & WSAS!=., clear

    drop if time3==1

    * define WHO-5 weights from response date relative to March 11
    ge datedif=int_dato-mdy(3,11,2020)
    replace datedif=datedif+1 if datedif>=0
    // +1 so those responded on March 11 counts as just after
    ta datedif
    ge w_WHO5=15
    replace w_WHO5=datedif if inrange(datedif,0,14)
    ta w_WHO5
    replace w_WHO5=w_WHO5/15
    ta w_WHO5

    qui reg WSAS_10 time1 time2 $X1 [aw=w_WHO], nocons vce(cl pnr)
    test time1=time2
    local F=r(F)
    local df=r(df)
    local p=r(p)
    margins, atmeans at(time1==1 time2==0) post
    ge b1=_b[_cons]
    ge ll1=b1-1.96*_se[_cons]
    ge ul1=b1+1.96*_se[_cons]
    qui reg WSAS_10 time1 time2 $X1 [aw=w_WHO], nocons vce(cl pnr)
    margins, atmeans at(time1==0 time2==1) post
    ge b2=_b[_cons]
    ge ll2=b2-1.96*_se[_cons]
    ge ul2=b2+1.96*_se[_cons]
    keep b1 ll1 ul1 b2 ll2 ul2
    keep if _n==1
    ge n=1
```

```
    reshape long b ll ul, i(n) j(time)
    drop n

    * export for plotting in R
    export excel using FigureA7_weighted.xlsx, replace firstrow(variables)

    * stata plot
    two bar b time, fcolor(white) lcolor(gs1) barwidth(.45) || ///
        rcap ul ll time, lcolor(gs1) ///
        xlabel(1 "Before lockdown" 2 "During lockdown March") ///
        xtitle("") ///
        ylabel(0(.1).4) ///
        ytitle("") ///
        legend(off) ///
        title("March 12 - March 25 downweighted") ///
        name(FigA7_w_all, replace)

    * rerun main results
    use pnr time* WHO5 WSAS WSAS_10 $X1 ///
        using panel_unbalanced_march_july.dta if WHO5!=. & WSAS!=., clear

    drop if time3==1

    qui reg WSAS_10 time1 time2 $X1, nocons vce(cl pnr)
    test time1=time2
    local F=r(F)
    local df=r(df)
    local p=r(p)
    margins, atmeans at(time1==1 time2==0) post
    ge b1=_b[_cons]
    ge ll1=b1-1.96*_se[_cons]
    ge ul1=b1+1.96*_se[_cons]
    qui reg WSAS_10 time1 time2 $X1, nocons vce(cl pnr)
    margins, atmeans at(time1==0 time2==1) post
    ge b2=_b[_cons]
    ge ll2=b2-1.96*_se[_cons]
    ge ul2=b2+1.96*_se[_cons]
    keep b1 ll1 ul1 b2 ll2 ul2
    keep if _n==1
    ge n=1
    reshape long b ll ul, i(n) j(time)
    drop n

    * export for plotting in R
    export excel using FigureA7_main.xlsx, replace firstrow(variables)

    * stata plot
    two bar b time, fcolor(white) lcolor(gs1) barwidth(.45) || ///
        rcap ul ll time, lcolor(gs1) ///
        xlabel(1 "Before lockdown" 2 "During lockdown March") ///
        xtitle("") ///
        ylabel(0(.1).4) ///
        ytitle("") ///
        legend(off) ///
        title("All: Main results") ///
        name(FigA7_main_all, replace)

    foreach v in Single Couple NoChildren Children{
      use pnr int_dato time* WHO5 WSAS WSAS_10 `v' $X1 ///
          using panel_unbalanced_march_july.dta if WHO5!=. & WSAS!=., clear

      drop if time3==1
      keep if `v'==1

      * define WHO-5 weights from response date relative to March 11
      ge datedif=int_dato-mdy(3,11,2020)
      replace datedif=datedif+1 if datedif>=0
      // +1 so those responded on March 11 counts as just after
      ta datedif
      ge w_WHO5=15
      replace w_WHO5=datedif if inrange(datedif,0,14)
      ta w_WHO5
      replace w_WHO5=w_WHO5/15
      ta w_WHO5

```

```
        qui reg WSAS_10 time1 time2 $X1 [aw=w_WHO], nocons vce(cl pnr)
        test time1=time2
        local F=r(F)
        local df=r(df)
        local p=r(p)
        margins, atmeans at(time1==1 time2==0) post
        ge b1=_b[_cons]
        ge ll1=b1-1.96*_se[_cons]
        ge ul1=b1+1.96*_se[_cons]
        qui reg WSAS_10 time1 time2 $X1 [aw=w_WHO], nocons vce(cl pnr)
        margins, atmeans at(time1==0 time2==1) post
        ge b2=_b[_cons]
        ge ll2=b2-1.96*_se[_cons]
        ge ul2=b2+1.96*_se[_cons]
        keep b1 ll1 ul1 b2 ll2 ul2
        keep if _n==1
        ge n=1
        reshape long b ll ul, i(n) j(time)
        drop n

        * export for plotting in R
        export excel using FigureA7_weighted_`v'.xlsx, replace firstrow(variables)

        * stata plot
        two bar b time, fcolor(white) lcolor(gs1) barwidth(.45) || ///
          rcap ul ll time, lcolor(gs1) ///
          xlabel(1 "Before lockdown" 2 "During lockdown March") ///
          xtitle("") ///
          ylabel(0(.1).4) ///
          ytitle("") ///
          legend(off) ///
          title("March 12 - March 25 downweighted") ///
          name(FigA7_w_`v', replace)

        * rerun main results
        use pnr time* WHO5 WSAS WSAS_10 `v' $X1 ///
          using panel_unbalanced_march_july.dta if WHO5!=. & WSAS!=., clear

        drop if time3==1
        keep if `v'==1

        qui reg WSAS_10 time1 time2 $X1, nocons vce(cl pnr)
        test time1=time2
        local F=r(F)
        local df=r(df)
        local p=r(p)
        margins, atmeans at(time1==1 time2==0) post
        ge b1=_b[_cons]
        ge ll1=b1-1.96*_se[_cons]
        ge ul1=b1+1.96*_se[_cons]
        qui reg WSAS_10 time1 time2 $X1, nocons vce(cl pnr)
        margins, atmeans at(time1==0 time2==1) post
        ge b2=_b[_cons]
        ge ll2=b2-1.96*_se[_cons]
        ge ul2=b2+1.96*_se[_cons]
        keep b1 ll1 ul1 b2 ll2 ul2
        keep if _n==1
        ge n=1
        reshape long b ll ul, i(n) j(time)
        drop n

        * export for plotting in R
        export excel using FigureA7_main_`v'.xlsx, replace firstrow(variables)

        * stata plot
        two bar b time, fcolor(white) lcolor(gs1) barwidth(.45) || ///
          rcap ul ll time, lcolor(gs1) ///
          xlabel(1 "Before lockdown" 2 "During lockdown March") ///
          xtitle("") ///
          ylabel(0(.1).4) ///
          ytitle("") ///
          legend(off) ///
          title("`v': Main results") ///
          name(FigA7_main_`v', replace)
      }
```

```

    * combine to produce Figure A7
    graph combine FigA7_main_all FigA7_w_all ///
                  FigA7_main_Single FigA7_w_Single ///
                  FigA7_main_Couple FigA7_w_Couple ///
                  FigA7_main_NoChildren FigA7_w_NoChildren ///
                  FigA7_main_Children FigA7_w_Children ///
                , iscale(1) cols(2) ///
                altshrink title("At risk of depression or stress (WHO-5 < 50)")
    graph export FigureA7.png, replace


  ////////////////////////////////////////////////////////////////////////////////
  //  Table A9: Summary of results from individual fixed effects specification
  ////////////////////////////////////////////////////////////////////////////////

    * WHO5 < 50
    use pnr int_dato time* WHO5 WHO5_50 WSAS WSAS_10 $X1 $X2 ///
        using panel_unbalanced_march_july.dta if WHO5!=. & WSAS!=., clear
    xtset pnr time

    xtreg WHO5_50 time2 time3 $X1, fe vce(cl pnr)
    eststo WHO5_FE
    xtreg WHO5_50 time2 time3 $X1, fe vce(cl pnr), if Single==1
    eststo WHO5_FE_single
    xtreg WHO5_50 time2 time3 $X1, fe vce(cl pnr), if Single==0
    eststo WHO5_FE_couple
    xtreg WHO5_50 time2 time3 $X1, fe vce(cl pnr), if Children==0
    eststo WHO5_FE_nokids
    xtreg WHO5_50 time2 time3 $X1, fe vce(cl pnr), if Children==1
    eststo WHO5_FE_kids

    * WSAS > 10
    xtreg WSAS_10 time2 time3 $X1, fe vce(cl pnr)
    eststo WSAS_FE
    xtreg WSAS_10 time2 time3 $X1, fe vce(cl pnr), if Single==1
    eststo WSAS_FE_single
    xtreg WSAS_10 time2 time3 $X1, fe vce(cl pnr), if Single==0
    eststo WSAS_FE_couple
    xtreg WSAS_10 time2 time3 $X1, fe vce(cl pnr), if Children==0
    eststo WSAS_FE_nokids
    xtreg WSAS_10 time2 time3 $X1, fe vce(cl pnr), if Children==1
    eststo WSAS_FE_kids

    * print Table A9
    esttab WHO5_FE WHO5_FE_single WHO5_FE_couple WHO5_FE_nokids WHO5_FE_kids ///
         using TableA9.rtf, se replace mtitles ///
         keep(_cons time2 time3) order(_cons time2 time3) nonotes
    esttab WSAS_FE WSAS_FE_single WSAS_FE_couple WSAS_FE_nokids WSAS_FE_kids ///
         using TableA9.rtf, se append mtitles ///
         keep(_cons time2 time3) order(_cons time2 time3)


  ////////////////////////////////////////////////////////////////////////////////
  //  Figure A8: Proportion of respondents at risk of depression or stress
  //             according to the WHO-5, comparing main results for respondents
  //             with children living at home by age of the children
  ////////////////////////////////////////////////////////////////////////////////

    * WHO-5 < 50
    use pnr int_dato time* WHO5 WHO5_50 WSAS $X1 $X2 Infants ///
        using panel_unbalanced_march_july.dta if WHO5!=. & WSAS!=., clear

    drop if time3==1
    keep if Children==1

    foreach v in Main Infants Toddlers Schoolers{
      preserve
      if "`v'"=="Main" local f=" "
      if "`v'"=="Infants" local f=", if inrange(Infants,1,3)"
      if "`v'"=="Toddlers" local f=", if inrange(Toddlers,1,3)"
      if "`v'"=="Schoolers" local f=", if inrange(Schoolers,1,3)"
      qui reg WHO5_50 time1 time2 $X1, nocons vce(cl pnr) `f'
      test time1=time2
      local F=r(F)
```

```
    local df=r(df)
    local p=r(p)
    margins, atmeans at(time1==1 time2==0) post
    ge b1=_b[_cons]
    ge ll1=b1-1.96*_se[_cons]
    ge ul1=b1+1.96*_se[_cons]
    qui reg WHO5_50 time1 time2 $X1, nocons vce(cl pnr) `f'
    margins, atmeans at(time1==0 time2==1) post
    ge b2=_b[_cons]
    ge ll2=b2-1.96*_se[_cons]
    ge ul2=b2+1.96*_se[_cons]
    keep b1 ll1 ul1 b2 ll2 ul2
    keep if _n==1
    ge n=1
    reshape long b ll ul, i(n) j(time)
    drop n

    * export for plotting in R
    export excel using FigureA8_`v'.xlsx, replace firstrow(variables)

    * stata plot
    two bar b time, fcolor(white) lcolor(gs1) barwidth(.45) || ///
      rcap ul ll time, lcolor(gs1) ///
      xlabel(1 "Before lockdown" 2 "During lockdown March") ///
      xtitle("") ///
      ylabel(0(.1).4) ///
      ytitle("Share") ///
      legend(off) ///
      title("`v'") ///
      name(A8_`v', replace)
    restore
  }

  * graph combine to produce Figure A8
  graph combine A8_Main A8_Infants A8_Toddlers A8_Schoolers ///
        , iscale(1) cols(2) altshrink ///
        title("At risk of depression or stress (WHO-5 < 50)")
  graph export FigureA8.png, replace


////////////////////////////////////////////////////////////////////////////////
//  Figure A9: Proportion of respondents experiencing significant functional
//             impairment according to the WSAS, comparing main results for
//             respondents with children living and home and results by age
//             of the children
////////////////////////////////////////////////////////////////////////////////

  * WSAS > 10
  use pnr int_dato time* WHO5 WSAS WSAS_10 $X1 $X2 Infants ///
      using panel_unbalanced_march_july.dta if WHO5!=. & WSAS!=., clear

  drop if time3==1
  keep if Children==1

  foreach v in Main Infants Toddlers Schoolers{
    preserve
    if "`v'"=="Main" local f=" "
    if "`v'"=="Infants" local f=", if inrange(Infants,1,3)"
    if "`v'"=="Toddlers" local f=", if inrange(Toddlers,1,3)"
    if "`v'"=="Schoolers" local f=", if inrange(Schoolers,1,3)"
    qui reg WSAS time1 time2 $X1, nocons vce(cl pnr) `f'
    test time1=time2
    local F=r(F)
    local df=r(df)
    local p=r(p)
    margins, atmeans at(time1==1 time2==0) post
    ge b1=_b[_cons]
    ge ll1=b1-1.96*_se[_cons]
    ge ul1=b1+1.96*_se[_cons]
    qui reg WSAS time1 time2 $X1, nocons vce(cl pnr) `f'
    margins, atmeans at(time1==0 time2==1) post
    ge b2=_b[_cons]
    ge ll2=b2-1.96*_se[_cons]
    ge ul2=b2+1.96*_se[_cons]
    keep b1 ll1 ul1 b2 ll2 ul2
```

```
    keep if _n==1
    ge n=1
    reshape long b ll ul, i(n) j(time)
    drop n

    * export for plotting in R
    export excel using FigureA9_`v'.xlsx, replace firstrow(variables)

    * stata plot
    two bar b time, fcolor(white) lcolor(gs1) barwidth(.45) || ///
      rcap ul ll time, lcolor(gs1) ///
      xlabel(1 "Before lockdown" 2 "During lockdown March") ///
      xtitle("") ///
      ylabel(0(.1).4) ///
      ytitle("Share") ///
      legend(off) ///
      title("`v'") ///
      name(A9_`v', replace)
    restore
  }

  * graph combine to produce Figure A9
  graph combine A9_Main A9_Infants A9_Toddlers A9_Schoolers ///
        , iscale(1) cols(2) altshrink ///
        title("Significantly functionally impaired (WSAS > 10)")
  graph export FigureA9.png, replace


////////////////////////////////////////////////////////////////////////////////
//  That's it!
    clear all
////////////////////////////////////////////////////////////////////////////////
```
